# STW-MD: a novel spatio-temporal weighting and multi-step decision tree method for considering spatial heterogeneity in brain gene expression data

**DOI:** 10.1093/bib/bbae051

**Published:** 2024-02-21

**Authors:** Shanjun Mao, Xiao Huang, Runjiu Chen, Chenyang Zhang, Yizhu Diao, Zongjin Li, Qingzhe Wang, Shan Tang, Shuixia Guo

**Affiliations:** Department of Statistics, Hunan University, Shijiachong Road, Changsha 410000, China; Department of Statistics, Hunan University, Shijiachong Road, Changsha 410000, China; Department of Statistics, Hunan University, Shijiachong Road, Changsha 410000, China; Department of Statistics, Hunan University, Shijiachong Road, Changsha 410000, China; Department of Statistics, Hunan University, Shijiachong Road, Changsha 410000, China; Central University of Finance and Economics; Shanghai Institute for Advanced Studies, University of Science and Technology of China; Department of Statistics, Hunan University, Shijiachong Road, Changsha 410000, China; MOE-LCSM, School of Mathematics and Statistics, Hunan Normal University, Lushan Road, Changsha 410000, China

**Keywords:** Alzheimer’s disease, brain development, spatial heterogeneity, spatial-temporal weighting, multi-step decision trees

## Abstract

Gene expression during brain development or abnormal development is a biological process that is highly dynamic in spatio and temporal. Previous studies have mainly focused on individual brain regions or a certain developmental stage. Our motivation is to address this gap by incorporating spatio-temporal information to gain a more complete understanding of brain development or abnormal brain development, such as Alzheimer’s disease (AD), and to identify potential determinants of response. In this study, we propose a novel two-step framework based on spatial-temporal information weighting and multi-step decision trees. This framework can effectively exploit the spatial similarity and temporal dependence between different stages and different brain regions, and facilitate differential gene analysis in brain regions with high heterogeneity. We focus on two datasets: the AD dataset, which includes gene expression data from early, middle and late stages, and the brain development dataset, spanning fetal development to adulthood. Our findings highlight the advantages of the proposed framework in discovering gene classes and elucidating their impact on brain development and AD progression across diverse brain regions and stages. These findings align with existing studies and provide insights into the processes of normal and abnormal brain development.

## INTRODUCTION

Human brain development is a dynamic and highly regulated biological process (BP) that unfolds over a protracted period [1–3]. Precise regulation of gene expression is crucial for brain development and function, which undergoes dynamic changes from fetal development to adulthood [4]. With a focus on brain development in adolescence, [5] had investigated the gene regulation processes responsible for the significant maturation of brain development from childhood to adulthood. Studying gene expression trajectories during brain development can provide insights into advanced behavioral functions in humans, such as the abilities of learning and memory [6, 7]. Furthermore, abnormal brain development at different stages can contribute to various serious diseases. These include autism spectrum disorder (ASD) with early childhood onset [8], schizophrenia with late adolescence or early adulthood [9], and Alzheimer’s disease (AD) in older age groups [10]. These disorders of brain development, with a high medical burden, have attracted global attention. Among them, the progress of AD is a continuous process from preclinical disease, mild cognitive and/or behavior disorder, dementia and AD [11–13]. Thus, the study of differential gene expression in different periods of brain development or different stages of related diseases helps to promote the understanding of brain development and disease mechanism.

In essence, the gene expression data related to brain development or abnormal development can be summarized as a kind of spatial-temporal dynamic evolution data, that is, there are multiple different periods or stages, and the gene expression data of different brain regions. Advancements in modern biotechnology have enabled the acquisition of richer and more detailed spatial-temporal data of the brain. For example, [14] developed Brain EXPression Database, a comprehensive database that provides gene expression data across specific brain regions, age ranges and genders. Different gene expression levels in various stages and regions of brain development may have distinct regulatory effects. [15] stated that the transcriptional heterogeneity of human microglia varies by brain region and aging. Furthermore, insights from AD highlight the importance of investigating regional alterations in brain structure and functionality for elucidating disease pathogenesis. [16] found that gray matter density in the hippocampus, amygdala, and basal forebrain decreased faster in AD patients than in healthy control subjects, and subcortical regions also decreased faster than neocortical brain regions. Hence, it is crucial to understand gene regulation across multiple brain regions and developmental stages in order to gain mechanistic insights into brain development and abnormal development.

The spatio-temporal data of brain development mainly involve two questions: one is to study which genes play a role in brain development or developmental abnormalities. The other is to study which brain regions and during what developmental time periods these genes play a role. Several approaches have been developed to address the temporal and spatial dynamics data of brain development and/or abnormal development. [17] proposed a two-step approach based on Markov random fields to effectively utilize the spatio-temporal information embedded in brain regions. This approach improves the identification of differentially expressed genes (DEGs) during brain development. [18] investigated paired DNAm and transcriptome data from four brain regions in AD patients, and linked methylation differences to local gene dysregulation through brain region stratification analysis and cross-region differential methylation analysis. [19] constructed a transcriptome-based weighting polygenic risk score for each brain region and the MultiXcan statistical method was used to integrate the results of transcriptome wide association studies in these 13 brain regions, which provided additional information for identifying individuals at high risk for AD [20]. However, the above methods either solely focus on the brain region factor without considering the time scale factor, or they consider both but ignore the gene heterogeneity during brain development, that is, the expression level of the same gene is not consistent across different regions of the brain. Regardless of the clinical presentation, both neurological and psychiatric disorders demonstrate high individual and regional heterogeneity in gene expression patterns [21]. For example, in AD patients, gene expression patterns in microglia exhibit high dynamics and heterogeneity due to epigenetic modifications and non-coding RNA regulation [22]. Therefore, it is necessary to address the above issues and consider the heterogeneity of genes when studying the spatio-temporal dynamics data of brain development.

Previous studies of gene expression data for different stages of brain development or brain diseases have either considered only the temporal nature of different developmental stages, or only the spatial dimension of different brain regions. Concomitantly, owing to the inherent heterogeneity within gene expression data, namely the substantial diversities in functional contributions of genes in distinct brain regions over the course of development or disease progression, a direct analysis of differential gene expression may not adequately account for these sources of variability. Considering the spatio-temporal dynamics of gene expression data during brain development or abnormal development, a two-step modeling framework based on spatio-temporal information weighting and multi-step decision trees (STW-MD) is proposed in this paper. An advantage of this framework is its capability for large-scale gene discovery associated with specific stages of both brain development and abnormal development. Concurrently, as an efficient and interpretable method, the framework can well adapt to some existing differential gene analysis methods and clustering algorithms. The methodology is described in Section 2. Section 3 demonstrates the application of the proposed framework to AD datasets and neurodevelopmental data, presenting key insights generated. Finally, Section 4 concludes the paper.

## MATERIALS AND METHODS

### Datasets

To demonstrate the effectiveness of the proposed method under the spatio-temporal data of the brain, this study intends to conduct research based on two datasets: the AD dataset and the brain development dataset. The former was obtained from the Mount Sinai Medical Center Brain Bank, which included data from 85 samples of AD patients [23]. Each AD sample contained data on the expression levels of 18 431 genes in 19 brain regions from early, middle to late stages. The staging criteria primarily relied on the Clinical Dementia Rating Scale. The brain development dataset was obtained from the BrainSpan database [24], which was collected from 1340 tissue samples of 57 developmental and postmortem adult brains and contains gene expression data of 16 brain regions at 15 stages of brain development from embryonic development to late adulthood. After excluding non-coding genes and lowly expressed genes, a total of 15 210 genes were retained as the background for further analysis. Details and features of the two datasets are provided in Supplementary Material S1.

Suppose different periods or stages of brain development are defined as $(0, 1,2,\cdots ,T)$. Let $y_{t,i,r,g}$ denote the observed gene expression value for gene $g$ in the $i$th samples in brain region ${r}$ and period $t$, and let $\mathbf{N}=(N_{0},N_{1},\ldots ,N_{T})$ denote the number of samples in different periods.

### Methods

#### Weighting brain regions considering genetic heterogeneity

**Figure 1:**
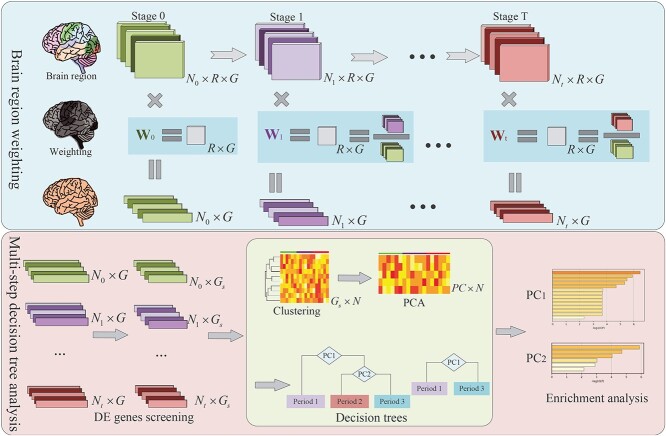
A Framework for Investigating Spatio-Temporal Dynamics of Gene Expression during Brain Development. The proposed model framework is categorized into two distinct components: brain region weighting (top panel, light blue area) and multi-step decision tree analysis (bottom panel, light red area). The initial layer of the brain region weighting section encompasses brain gene expression samples derived from various evolutionary periods across brain development. Each distinct sample is visually represented as an $R \times G$ matrix block, where the rows and columns correspond to diverse brain regions and genes, respectively. Subsequently, the second layer involves the calculation of weights assigned to distinct brain regions. These weights are primarily derived from the comparative analysis of sample information between the current period’s brain regions and the period $0$. The third layer illustrates the results of weighted gene expression, with each block denoting the gene expression of an individual sample. The multi-step decision tree analysis was divided into three main parts: the DE gene screening process, the clustering and decision tree process (light green area) and the enrichment analysis process.

This paper introduces a novel weighting method for analyzing spatio-temporal dynamic expression data. The proposed method constructs weights based on the differential expression information observed at different stages. These weights are then applied to all brain regions within the corresponding stages, resulting in a reduction of spatial dimensionality. The graphical representation of the weighting method is depicted in the light blue section of Figure 1.

Conventional weighting approaches, as outlined in previous studies [25], typically utilize dimensionality reduction techniques similar to arithmetic averaging to weight the spatio-temporal gene expression data. However, these strategies often result in substantial loss of the original information and fail to adequately address the challenge of gene heterogeneity across different brain regions. To address these limitations, the Fold Change (FC) was utilized as the weighting factor to integrate gene expression profiles across different brain regions in every stage [26, 27]. The FC was principally computed from the ratio of average gene abundances between two different stages, and each gene was corresponding to different weights in different stages and different brain regions. By selecting FC as the weighting metric, inter-genic, inter-regional and temporally divergent variability could be holistically encompassed.

The weight FC of brain region $r$ in period $t$ is shown as follows: 


(1) \begin{align*}& FC_{t, r, g}=\begin{cases} \frac{\sum_{i_{t}=1}^{N_{t}} y_{t,i_{t},r,g}/N_{t}}{\sum_{i_{0}=1}^{N_{0}} y_{0,i_{0},r,g}/N_{0}} & \textrm{if}\ t \geq 1\\ 1 & \textrm{if}\ t = 0 \end{cases}\end{align*}

where $y_{t,i_{t},r,g}$ and $y_{0,i_{0},r,g}$ are the gene expression values, and $N_{t}$ and $N_{0}$ represent the sample size in the corresponding period. Meanwhile, period $t=0$ can be considered as the control group of period $t \geq 1$, for example, when studying gene expression analysis in the onset stage of AD, period $t=0$ represents the gene expression level in healthy individuals.

FC value is usually used to identify DEGs between two diseases, but $\log _{2} FC$ is more suitable to deal with asymmetric expression differences than FC value, and can provide a more accurate measure of genes with low expression [28]. Here, we use the $\log _{2} FC$ value as the weight of brain regions, which can well take into account the differences in developmental evolution of different genes in different brain regions. Hence, the brain region gene expression of sample $i_{t}$ in period $t$ was weighted as follows: 


(2) \begin{align*}& \begin{aligned} y_{t,i_{t},g}^{FC} = \frac{\sum_{r_{t}=1}^{R} y_{t,i_{t},r_{t},g} \times \log_{2} FC_{t, r_{t}, g}} {\sum_{r_{t}=1}^{R} \log_{2} FC_{t, r_{t}, g}} \end{aligned}\end{align*}

where $y_{t,i_{t},g}^{FC}$ represents the weighted gene expression of gene $g$ corresponding to individual $i_{t}$ in period $t$ over $R$ brain regions. The weighted gene expression considers gene heterogeneity and expression differences across brain regions, enabling subsequent analysis using existing methods.

Meanwhile, it is crucial to consider that the same gene can simultaneously exhibit both upregulation and downregulation across different brain regions during the same developmental stage or even across different stages [29]. This poses a challenge when directly applying weights, as positive and negative values can counterbalance each other, resulting in the neutralization of differential gene expression. To overcome this issue, the previously defined weights need further adjustments to effectively differentiate gene expression levels across different stages after weighting. Based on the FC threshold commonly used in the literature for screening differential genes, the weight threshold in this study was set at 1.5 [30, 31]. Therefore, when the calculated FC values between genes in different stages meet $|\log _{2} FC |> \log _{2} 1.5$ (equivalent to $FC = 1.5, FC = 1/1.5$), we consider the gene to have a significant difference in expression between different stages. In this case, the adjusted weights are shown as follows: 


(3) \begin{align*}& \log_{2} FC_{t, r, g}^{ad} \triangleq \begin{cases} -\log_{2} FC_{t, r, g} & \textrm{ if } \log_{2} FC_{t, r, g}< - \log_{2} 1.5 \\ \log_{2} FC_{t, r, g} & \textrm{ if } \log_{2} FC_{t, r, g} \geq \log_{2} 1.5 \\ 0 & \text{others.} \end{cases}\end{align*}

where $\log _{2} FC_{t, r, g}^{ad}$ represents the adjusted weight, $\log _{2} F C_{t, r, g} \geq \log _{2} 1.5$ indicates up-regulated genes, $\log _{2} F C_{t, r, g}< - \log _{2} 1.5$ indicates down-regulated genes, and the others are considered insignificant. For the case that the gene is not significant in the brain region, if its weight is not set to 0, the significant expression level of the gene will be diluted by the insignificant DEG levels in other brain regions during the weighting process, thus causing an unreasonable situation. More details are discussed can be found in Supplementary Material S2.

To mitigate the impact of substantial variations in gene expression across different brain regions, a standardization step was performed on the gene expression values within the matrix. Consequently, the normalized expression level of gene $g$ at period $t$ can be computed as follows: 


(4) \begin{align*}& \begin{aligned} y_{t,i,r,g}^{norm}=\frac{y_{t,i,r,g}-\underset{r \in (1,2,\dots,R)}{\min} y_{t,i,r,g}}{\underset{r \in (1,2,\dots,R)}{\max} y_{t,i,r,g} -\underset{r \in (1,2,\dots,R)}{\min} y_{t,i,r,g}} \times a + 1 \end{aligned}\end{align*}

where $y_{t,i,r,g}^{norm}$ represents the normalized result, $\underset{r \in (1,2,\dots ,R)}{\max } y_{t,i,r,g}$ and $\underset{r \in (1,2,\dots ,R)}{\min } y_{t,i,r,g} $ represent the maximum and minimum gene expression levels in $R$ brain regions of period $t$, respectively, and $a$ is a constant. Specifically, we set $a = 3$ for normalization to maximize the differences in gene expression across stages, constraining the values within the range of [1,4].

To concisely recapitulate the sequential steps comprising the present methodology, they may be delineated as follows:

- Step 1: normalize $\boldsymbol{y}$ to $\boldsymbol{y}^{norm}$ by Formula (4);
- Step 2: calculated adjusted FC value by Formula (3);
- Step 3: calculate weighted gene expression by Formula (2).

#### Multi-step decision tree based on gene clusters

In the previous section, gene expression data of individuals at different periods were obtained by weighting the data of brain regions. In the next step, some common differential gene analysis methods can be combined to identify the key genes classes that distinguish different periods of brain development or disease progression. In this paper, a strategy of integrating multiple methods was used for analysis, that is, a multi-step decision tree method was used for differential gene expression analysis. The algorithm for the multi-step decision tree is delineated as follows (refer to the pink section of Figure 1):

- Step 1: screening DEGs. In this paper, the widely recognized Limma model was predominantly employed for this purpose [32, 33]. The selection of DEGs can also be regarded as an initial step of dimension reduction.
- Step 2: cluster analysis and matrix factorization. The DEGs screened above were grouped into several gene classes by cluster analysis, such as tSNE or UMAP combined with K-means clustering methods [34]. Additionally, the principal component analysis (PCA) [35] was utilized to extract the principal components (PCs) of each gene class, leading to quadratic dimension reduction and enhancing the interpretability of the subsequent decision tree model.
- Step 3: The decision tree model. The PCs of the above gene classes were used in the CART decision tree model to identify the key gene classes that distinguished AD patients at different stages [36].
- Step 4: Gene Set Enrichment Analysis. GO or KEGG enrichment analysis [37] was used to analyze the relationship between the above key gene classes and brain development or disease progression.

## RESULTS

### Exploring the influential factors of AD

#### Weighting results for DEGs based on FC values

**Figure 2:**
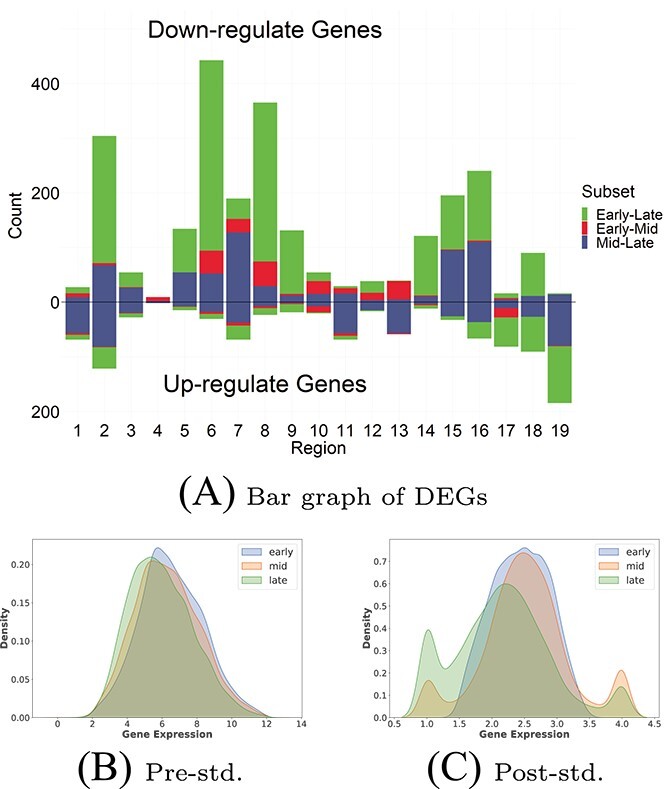
Bar plots and gene distributions plots. (**A**) represents a bar graph of level grouping of DEGs in 19 brain regions. The bars in the upper and lower halves represent the number of down-regulated and up-regulated genes, respectively. The different colors indicate the DEGs between pairs at various stages of AD. (**B**) and (**C**) show the gene distributions before and after normalization (Pre-std. and Post-std.), respectively.

In the research of AD data, it is often observed that the expression levels of most genes do not exhibit significant differences across different disease stages. Consequently, calculating weights for these genes may not be worthwhile. Therefore, this paper utilized the Limma model to perform an initial screening of DEGs at various time stages within each brain region. Figure 2(A) displays bar graphs depicting the number of up-regulated and down-regulated genes in different brain regions of the AD dataset. Notably, the differential expression of genes was primarily observed in the early-late and middle-late groups. After pooling the DEGs in each brain region, the number of significantly up-regulated genes (458) was significantly lower than that of significantly down-regulated genes (1830). Taking the union of all DEGs from the 19 brain regions, we obtained a total of 1014 DEGs.

Using the adjusted FC value, the DEGs in the 19 brain regions of AD patients were weighted after standardization. Figure 2(B and C) displays the weighted gene distributions before and after normalization. It is evident that there is a more pronounced difference between groups at different stages in AD patients, which is advantageous for subsequent genetic analyses. Details are discussed and illustrated in Supplementary Material S3.

#### Differential gene expression analysis after weighting

Differential gene expression analysis was performed on the weighted gene expression data. A total of 65 DEGs (37 up-regulated and 28 down-regulated genes) were identified in the early and middle stages, 100 DEGs (25 up-regulated and 75 down-regulated genes) in the early and late stages, and 136 DEGs (24 up-regulated and 112 down-regulated genes) in the middle and late stages. Overall, the expression levels of the DEGs exhibited a downward trend. Combining the DEGs from these three groups resulted in a total of 197 DEGs.

**Figure 3:**
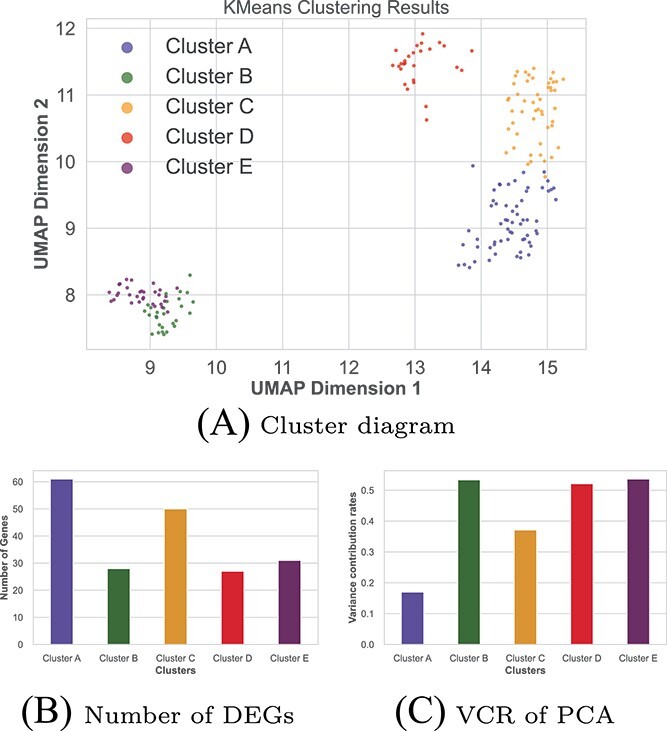
The result of the multi-step decision tree algorithm. The first two panels are the clustering results and the number of DEGs in each category, and the last panel is the VCR of the $PC_{1}$ after PCA for each cluster gene.

Figure 3(A and B) present the results of the cluster analysis of DEGs, where it is appropriate to divide them into five classes based on certain criteria of kmeans and defined as ClusterA, ClusterB, ClusterC, ClusterD and ClusterE, respectively. Panel (c) shows the variance contribution rate (VCR) of the first PC ($PC_{1}$) of the five groups of DEGs, among which the VCR of the $PC_{1}$ in each group of the five groups was mostly above 50%, indicating that the subsequent analysis based on the $PC_{1}$ was feasible.

**Figure 4:**
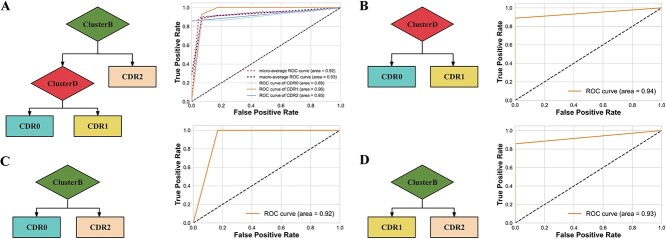
Early, mid and late decision trees and their classification ROC curves. Among them, diamonds represent taxonomic features (ClusterB and ClusterD), and rectangular blocks (early, mid and late) represent AD individuals in different stages.

Based on the $PC_{1}$ data of the above five groups of DEGs, Figure 4 illustrates the classification results of different stages using the decision tree model, along with their corresponding ROC curves. Figure 4(A) shows the decision tree of the early, middle and late stages, where the selection complexity coefficient $CP=0.05$ is utilized to trim the decision tree. The results show that the final classification accuracy of 20 times 5-fold cross validation (CV) is 88.41%, indicating that the model can well distinguish different stages of AD patients. Meanwhile, the pairwise decision trees of early, middle and late stage patients were constructed, and the prediction accuracy reached 90.58, 88.72 and 92.42%, respectively, as shown in the last three panels of Figure 4, in which the gene classes ClusterB and ClusterD played an important role in the classification of AD patients.

To ensure the robustness of results, we conducted 100 experiments where we constructed the four decision tree models using 80% of the individuals based on CV. Among these experiments, the decision tree in Figure 4 was observed to appear 78, 83, 79 and 96 times, respectively. In conclusion, our findings demonstrate the significance of the ClusterB gene class in association with late-stage AD patients, while highlighting the importance of the ClusterD gene class in distinguishing patients in the early and middle stages of AD.

Additionally, we compare our proposed weighting algorithm with two additional approaches: brain region averaging and imputation. The results show that our algorithm achieves notably better performance. For detailed information, please refer to Supplementary Material S3.4.

#### Enrichment analysis of ClusterB and ClusterD genes

**Figure 5:**
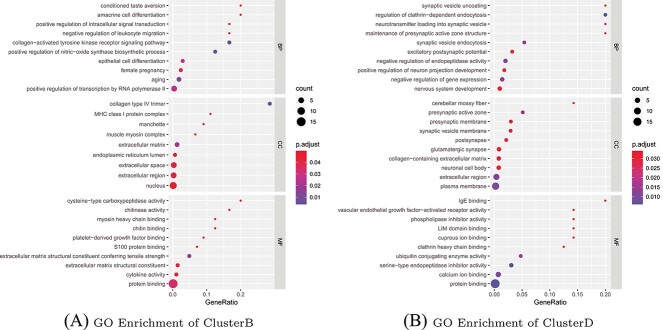
Bubble graphs depicting the top 10 most enriched functional categories of AD-related DEGs for two gene clusters, Cluster B and Cluster D. The analysis considers different functional categories, including BP, CC and MF, for each cluster.

To evaluate the significance of the two gene classes in distinguishing early, middle and late stages of AD, we conducted GO/KEGG enrichment analyses to examine their associations. Specifically, for the subsequent analysis, we primarily utilized the GO enrichment method provided by [38]. Figure 5 illustrates the enrichment bubble map for GO enrichment analysis of two classes of AD-related DEGs, ClusterB and ClusterD. The enrichment bubble map primarily displays the top 10 functional categories in BP, cellular component (CC) and molecular function (MF) that are most abundant in the two gene classes. Additionally, Table 1 presents the results of enrichment analysis for specific genes within these gene classes. Our analysis focuses on examining the characteristics of the functional categories in the two gene classes, as well as utilizing select genes to support our findings. More detailed results of the enrichment analysis can be found in the attached file.

**Table 1:** ClusterB and ClusterD gene enrichment analysis. Examples for specific genes within these gene classes.

Gene classes	GO term	Representative genes	Term	Adj. *P*-value
ClusterB	GO:0038063	COL4A1, COL4A2	Collagen-activated tyrosine kinase receptor signaling pathway	0.0049
	GO:0045944	NAMPT, FOS	Positive regulation of transcription by RNA polymerase II	0.0263
	GO:0016807	AQP1	Cysteine-type carboxypeptidase activity	0.0421
	GO:0061304	COL4A1	Retinal blood vessel morphogenesis	0.0421
	GO:0034356	NAMPT	NAD biosynthesis via nicotinamide riboside salvage pathway	0.0440
ClusterD	GO:0005515	MEF2C, ERC2, SNAP91, SH3GL2	Protein binding	0.0060
	GO:2000369	SNAP91, SH3GL2	Regulation of clathrin-dependent endocytosis	0.0060
	GO:0048488	SNAP91	Synaptic vesicle endocytosis	0.0155
	GO:0048786	ERC2, SLC17A7	Presynaptic active zone	0.0155
	GO:0030672	SLC17A7, SH3GL2	Synaptic vesicle membrane	0.0307
	GO:0048790	ERC2	Maintenance of presynaptic active zone structure	0.0340
	GO:1904753	MEF2C	Negative regulation of vascular associated smooth muscle cell migration	0.0340

The enrichment bubble plot of the ClusterB gene class (Figure 5A) reveals that its BP functional categories primarily revolve around the development and synaptic transmission processes of the nervous system, as well as regulatory mechanisms associated with neurotransmitters and synaptic structure. In terms of CC function, the emphasis lies on extracellular structures and intracellular organelles and complexes. Regarding MF, the focus is on the structural components of the extracellular matrix, protein binding, as well as functions related to enzymatic activity and cytokine activity. These functional categories align with the underlying pathological features of AD, specifically the ATN diagnostic framework [39, 40], suggesting the validity of the aforementioned enrichment analysis results.

As previously mentioned, we posit a notable association between the ClusterB gene class and AD patients in late-stage. In the late stages of AD, clinical manifestations become more pronounced, observable macroscopic alterations in brain biological traits occur, and microscopic protein detection can distinctly identify the advanced symptoms of AD [41]. Based on the results presented here, it is also possible to effectively distinguish patients with moderate-advanced AD by observing related traits controlled by genes in the ClusterB class. For example, [42] showed a potential association between gene AQP1 in astrocytes and A$\beta $ deposition in the AD brain, and senile plaques containing amyloid-beta peptide $A\beta _{1\text{-}42}$ are the major species present in the pathogenesis of AD [43]. Similarly, regulation of genes COL4A1, FOS and NAMPT had been linked to A$\beta $ deposition and potentially contributed to AD, with COL4A1 and FOS mainly acting through provoking inflammatory reactions [44–47].

The enrichment bubble plot of the ClusterD gene class (Figure 5B) reveals that its BP function involves multiple levels such as cell signal transduction and physiological regulation. The CC function focuses on neural cell structure, while the MF function involves different types of binding and regulatory activities. In contrast, the functional categories of the ClusterB gene class primarily focus on a certain aspect, whereas the functions of the ClusterD gene class cover a wider range.

Based on the analysis results of the decision tree, we assert a substantial association between the ClusterD gene class and AD patients in the early and middle stages. The results of ClusterD enrichment analysis (lower part of Table 1) revealed a key link between AD and the regulation of clathrin-dependent endocytosis and protein binding, that is, ClusterD class genes that can effectively distinguish between early and middle stage AD patients mainly control protein binding and neuronal synapse and neuronal cell-related metabolic processes. At this time, the clinical features of the patient are not obvious, but the internal metabolism of the brain has undergone microscopic changes [13, 48]. For example, as a neurodegenerative disease affecting cortical regions of the brain, abnormal presynaptic activity is also a potential feature affecting AD. Genes in the ClusterD, namely ERC2 and SLC17A7, are associated with presynaptic cell function [49, 50], while SNAP91 and SH3GL2 genes are involved in functions related to synaptic vesicles [51, 52]. In addition, [53] found that cerebral vascular smooth muscle cells were markedly reduced in AD patients, and the enrichment results of ClusterD gene class showed that MEF2C gene was involved in vascular associated smooth muscle cell migration, which verified the findings of this study [54]. [55] found that abnormal glycogen catabolism can also affect neuronal cell metabolism, which represents another potential pathogenic factor in AD, and this result also corresponds to the PGM2L1 gene in Table 1 [56]. More Details are illustrated in Supplementary Material S3.5.

### Repeatability analysis: human brain development

To further validate the reproducibility of the STW-MD method in our study, the analysis processes in human brain development were implemented identically. For convenience of analysis, the human brain development was categorized into three stages: fetal development (10 PCW $\leq $ Age < 38 PCW, AGE0), postnatal development (0 Y $\leq $ Age < 20 Y, AGE1), and adulthood (Age $\geq $ 20 Y, AGE2), where PCW for post-conceptional weeks and Y for postnatal years. Notably, data from at least three brain regions were obtained for 37 samples among the available samples.

A total of 13 479 DEGs were identified from a pool of 15 210 genes during the pre-weighted screening. This finding suggests that the expression levels of the majority of genes undergo significant changes throughout the three stages of brain development. Consequently, we selected the top 5% of pairwise $\log _{2} FC$ absolute values from each of the three periods as the weighted genes, resulting in a final set of 2003 DEGs after combining them. In the multi-step decision tree process, we conducted screening on the weighted data, resulting in the identification of 515 DEGs. The aforementioned genes were divided into four groups. Similarly, based on the results of the decision tree (Figure S.9), gene classes ClusterA, ClusterC and ClusterD were found to be associated with distinct stages of brain development.

The enrichment bubble plot in Figure S.10 clearly demonstrates distinct functional differences among the three gene classes. These enriched functions essentially reflect key features of brain development from fetal development to adulthood [57, 58]. For example, in terms of CC function, ClusterA is primarily associated with enveloped body-related structures such as coated nests and small bodies. ClusterC focuses on the nucleus and cytoplasm. ClusterD encompasses a wide range of intracellular and extracellular organelles, exhibiting both breadth and comprehensiveness.

In summary, based on the results of the decision tree (Figure S.9), cluster diagram (Figure S.8B) and enrichment bubble plot (Figure S.10), we suggest that ClusterA exhibits a strong association with AGE0, ClusterC differentiates the gene expression between AGE0 and AGE1, and ClusterD shows a notable association with AGE2. For example, ClusterA may be implicated in the healthy development and protection of the fetal brain (AGE0) [59]. [60] demonstrated the essential role of SOX9 as a determinant of cell fate during embryonic development. Its expression facilitates the differentiation of cells from all three germ layers into various specialized tissues and organs. More Details are illustrated in Supplementary Material S4.

## CONCLUSION

This paper focuses on the study of differential gene expression during dynamic and highly regulated brain development, including abnormal development. To effectively capture the spatial similarity and temporal dependence between different brain stages and regions, as well as address gene heterogeneity among various brain regions, this paper proposes a two-step modeling framework. The framework is based on spatio-temporal weighting and multi-step decision trees. This framework enables the analysis of differential gene expression in brain regions with high heterogeneity issues. Applying the framework to two distinct brain development datasets (the AD dataset and the brain development dataset), we discovered gene classes that exhibited significant correlations with specific developmental stages, demonstrating high consistency with existing studies. These findings offer valuable insights into the processes of brain development and abnormal development.

The major innovation of this study is to propose a new weighting method that combines spatial (all brain regions) and temporal (different stages of brain development) dimensions to explore the potential factors affecting or causing abnormal brain development. As mentioned earlier, the two-step modeling framework developed in this paper enables batch discovery of gene classes associated with specific stages of both AD and brain development. Moreover, it exhibits strong adaptability, allowing for the selection of more suitable indicators or algorithms based on the specific data requirements. For example, in the brain region weighting algorithm, in addition to the fold change weight (FC value) utilized in the current study, other quantitative metrics such as the $t$-statistic and $P$-value could potentially be evaluated individually or in conjunction as alternative weighting schemes. In addition, the current integration algorithm of the multi-step decision tree only considers the simple concatenation of common algorithms, which has a notable effect. However, each step of the integration algorithm offers numerous options for selection and combination that have not yet been explored. For instance, other prevalent differential gene screening methods (DESeq2 [61] and edgeR [62]), clustering algorithms (Seurat [63] and WGCNA [63]) and decision tree models (Random Forest [64] and XGBoost [65]) could be evaluated within the ensemble for a more comprehensive assessment.

This paper thoroughly investigates the two questions regarding spatiotemporal data of brain development raised in the Introduction. It primarily focuses on addressing the first question, taking into account the high heterogeneity observed among brain regions. Additionally, the second problem can be effectively solved through a straightforward backward derivation using the STW-MD method. Our method effectively addresses the issue of regional heterogeneity in an intuitive manner. Furthermore, it can be extended to incorporate other available data sources. For instance, the inclusion of functional magnetic resonance imaging data, single-cell data, and spatial transcriptome data can offer more comprehensive insights into the correlation and heterogeneity of brain regions [66–69]. This expansion enhances the rationality and reliability of the weighting process while facilitating a comprehensive analysis of gene expression heterogeneity within these regions. For a more detailed exploration, please refer to Supplementary Material S3.7. While the findings of this study align well with existing literature, it is important to note that our results do not specifically elucidate the functional role of individual genes. Instead, the emphasis lies on highlighting the associations between specific gene classes and distinct stages. Consequently, the interpretation of the biological significance of DEGs may be approached with a degree of simplicity. BPs are highly complex, with intricate connections between different genes. The study does not provide a macro perspective to interpret the results from a broader biologic perspective and may have overlooked other causal factors in AD that may be implicit in the results. These aspects will be further explored and discussed in future studies.

**Key Points**

- STW-MD is a two-step modeling framework based on spatio-temporal weighting and multi-step decision tree. It effectively captures spatial similarity and temporal dependency between different stages and brain regions, addressing the issue of genetic heterogeneity among different brain regions.
- STW-MD enables the batch discovery of gene classes associated with specific stages of both AD and brain development. It is an efficient and interpretable method.
- STW-MD demonstrates strong adaptability, allowing for the selection of more suitable weighting metrics or algorithms based on specific data requirements. It can effectively accommodate existing differential gene analysis methods and clustering algorithms.

## Supplementary Material

Revised2_Supplement_bbae051

Enrichment_analysis_2datasets_bbae051

## Data Availability

The code of STW-MD is available at https://github.com/tsnm1/STW-MD. The publicly available datasets from the Alzheimer’s disease and the brain development used in this study are described in the Supplementary Material S1.

## Author Biographies

**Shanjun Mao** is an assistant professor at Hunan University, specializing in Bayesian Data Analysis, Statistical Modeling and Computing, and Computational Biology/Bioinformatics.

**Xiao Huang** is a Master’s student at Hunan University, focusing on Statistical Modeling and Computing.

**Runjiu Chen** is an undergraduate student at Hunan University, with research interests in Biostatistics and Bioinformatics.

**Chenyang Zhang** is an undergraduate student at Hunan University, engaged in studies related to Biostatistics and Bioinformatics.

**Yizhu Diao** is an undergraduate student at Hunan University, exploring machine learning methodology and bioinformatics.

**Zongjin Li** is a Master’s student at Central University of Finance and Economics, specializing in Statistical Modeling and Computing.

**Qingzhe Wang** is an undergraduate student at Shanghai Institute for Advanced Studies, University of Science and Technology of China, focusing on Statistical Modeling and Computing.

**Shan Tang** is a PhD student at Hunan University, conducting research on statistical methods development.

**Shuixia Guo** is a Professor at Hunan Normal University, with expertise in the analysis and application of medical imaging Big Data.
