# Supplementary material for: STW-MD: a novel spatio-temporal weighting and multi-step decision tree method for considering spatial heterogeneity in brain gene expression data
Source: Brief Bioinform. 2024 Feb 21;25(2):bbae051. doi: 10.1093/bib/bbae051 (PMC10883420; doi:10.1093/bib/bbae051)
Supplement: Revised2_Supplement_bbae051 [file revised2_supplement_bbae051.pdf]

Supplement to "STW-MD: A Novel Spatio-Temporal Weighting and Multi-Step Decision Tree Method for Considering Spatial Heterogeneity in Brain Gene Expression Data"

Shanjun Mao[a], Xiao Huang[a], Runjiu Chen[a], Chenyang Zhang[a], Yizhu Diao[a], Zongjin Li[b], Qingzhe Wang[c], Shan Tang[a,*], Shuixia Guo[d,e,*]

[a] *Department of Statistics, Hunan University, Changsha 410079, China*
[b] *School of Statistics and Mathematics, Central University of Finance and Economics, Beijing 100081, China*
[c] *School of Management, Shanghai Institute for Advanced Studies, University of Science and Technology of China, Shanghai 201315, China*
[d] *MOE-LCSM, School of Mathematics and Statistics, Hunan Normal University, Changsha 410081, China*
[e] *Key Laboratory of Applied Statistics and Data Science, Hunan Normal University, College of Hunan Province, Changsha 410081, China*

Abstract

This supplement provides additional details and results for the Alzheimer's disease (AD) dataset and the brain development dataset.

In Section S1, we present the details and features of the AD dataset and the brain development dataset, highlighting their unique characteristics and relevant information. Section S2 focuses on the discussion and analysis of gene heterogeneous expression and adjusted fold change (FC) values. We explore the impact of gene heterogeneity and present the adjusted FC values. In Section S3, we provide detailed results for the AD dataset. Section S4 offers detailed results for the brain development dataset. In Section S5, we compare the DEGs between the two datasets in different methodological steps.

Overall, this supplement provides additional insights and results for the AD dataset and the brain development dataset, enhancing our understanding of gene

*Corresponding authors at: Department of Statistics, Hunan University, Shijiachong Road, Changsha 410000, China. E-mail: tangshanhaha@hnu.edu.cn (S.T.); MOE-LCSM, School of Mathematics and Statistics, Hunan Normal University, Lushan Road, Changsha 410000, China. E-mail: guoshuixia75@163.com (S.G.).

expression dynamics and the impact of gene heterogeneity on these datasets.

Contents

S1 Details and features of two dataset 2

S1.1 Details and features of the AD dataset 2

S1.2 Details and features of the brain development dataset 4

S1.3 Size and characteristics of the two datasets 5

S2 Analysis of gene heterogeneous expression 6

S3 Detailed results for the AD dataset 8

S3.1 Weighting results for DEGs based on FC values . . 8

S3.2 Comparison of different FC adjustment methods . 9

S3.3 The genetic heterogeneity of AD patients 13

S3.4 Introduction to weighting methods. 14

S3.5 Stability analysis of cluster analysis 15

S3.6 Results of other enrichment analyses 16

S3.7 Further discussion and analysis of brain heterogeneity 0

S4 Detailed results for brain development dataset 4

S4.1 Analysis results after Weighting 5

S4.2 Results of enrichment analyses 5

S5 Comparison of DEGs between the two datasets 10

S1. Details and features of two dataset

S1.1. Details and features of the AD dataset

According to the Clinical Dementia Rating Scale (CDR), this paper classified the AD patients into seven stages: CDR0, CDR0.5, CDR1, CDR2, CDR3, CDR4, CDR5. The number of samples in each stage is shown in Figure S.1. Meanwhile, Table S.1 shows the numbers and corresponding names of the 19 brain regions of the AD dataset.

However, AD patient samples did not have data in all brain regions, and individual AD patients only had gene expression data in 2 or 3 brain regions, while gene expression data in other brain regions were missing. Therefore, we

Table S.1: Brain region numbers and their corresponding names for AD dataset

Region	Structure	Structure acronym
Region1	Anterior Cingulate	AC
Region2	Caudate Nucleus	CN
Region3	Dorsolateral Prefrontal Cortex	DLPFC
Region4	Frontal Pole	FP
Region5	Hippocampus	Hippo
Region6	Inferior Frontal Gyrus	IFG
Region7	Inferior Temporal Gyrus	ITG
Region8	Middle Temporal Gyrus	MTG
Region9	Occipital Visual Cortex	OVC
Region10	Parahippocampal Gyrus	PHG
Region11	Posterior Cingulate Cortex	PCC
Region12	Precentral Gyrus	PreCG
Region13	Prefrontal Cortex	PFC
Region14	Putamen	Put
Region15	Superior Parietal Lobule	SPL
Region16	Superior Temporal Gyrus	STG
Region17	Temporal Pole	TP
Region18	Amygdala	Amyg
Region19	Nucleus Accumbens	NAcc

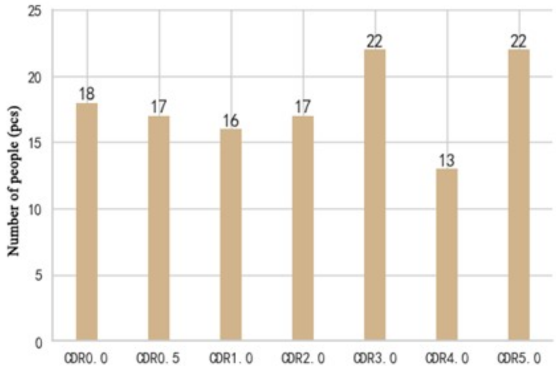

Figure S.1: Number of people in different stages of AD

deleted the patient samples with gene expression records in less than 3 brain regions, and finally retained 85 patient samples. The patients were also divided into three groups according to their AD severity (CDR): early group (containing CDR0, CDR0.5), middle group (containing CDR1, CDR2) and late group (containing CDR3, CDR4, CDR5). At the same time, the genes contained in the gene expression data of all brain regions were concatenated as a collection of study genes, and a total of 18431 related genes were obtained.

S1.2. Details and features of the brain development dataset

The brain development dataset is RNA sequencing and exome microarray microarray data, to analyze up to 16 cortical and subcortical structures throughout human brain development. Human brain microarray data to study dynamic changes in neurodevelopment. Chip data from 15 (pregnancy to adult) period of 16 brain regions. Table S.2 presents the brain region numbers and their corresponding names for the brain development data. But keep it simple, according to fetal development (< 38PCW, periods 3-7), postnatal development (< 20 Y, periods 8-12) and adulthood (≥ 20, periods 13-15) were divided into three periods.

The transcripts were filtered by the following two procedures. First, we filtered out non-coding genes, with 25167 protein coding genes retained based

on GRCh37 annotation (Ensembl release 75 from BioMart). We then filtered out protein coding genes with low expression level in the BrainSpan dataset, with 15210 genes remaining which have 80% of their reads per kilo million bases (RPKMs) greater than 0 and at least one RPKM greater than 1 across all samples in the BrainSpan dataset.

Table S.2: Brain region numbers and their corresponding names for brain development data

Region	Structure name	Structure acronym
Region1	primary auditory cortex	A1C
Region2	amygdala	AMY
Region3	cerebellar cortex	CBC
Region4	dorsolateral prefrontal cortex	DFC
Region5	hippocampus	HIP
Region6	posterior inferior parietal cortex	IPC
Region7	inferior temporal cortex	ITC
Region8	primary motor cortex	M1C
Region9	mediodorsal nucleus of the thalamus	MD
Region10	medial prefrontal cortex	MFC
Region11	orbital prefrontal cortex	OFC
Region12	primary somatosensory cortex	S1C
Region13	posterior superior temporal cortex	STC
Region14	striatum	STR
Region15	primary visual cortex	V1C
Region16	ventrolateral prefrontal cortex	VFC

S1.3. Size and characteristics of the two datasets

Table S.3 presents sample size information and feature details for both the AD dataset and the brain development dataset. These samples encompass the entire spectrum of cognitive and neuropathological disease severity in the absence of discernable non-AD neuropathology [1]. As shown in Table S.3, some basic information in the AD dataset and brain development dataset, such as

Sex, Age, and PH, have p-values from grouping test results greater than 0.05. This suggests that the average levels of these variables are not significantly different across various stages. Among them, the PMI represents the time that has elapsed since an individual's death. However, there is no clear correlation between the significance of this indicator and AD. On the other hand, variables like PLQ_Mn, NPrSum, and NTrSum, which are closely related to different stages of AD, exhibit p-values less than 0.05, indicating significant differences in the pathological characteristics of AD patients at distinct stages. It's worth noting that the brain development dataset is inherently grouped by age, resulting in significant differences between age groups.

Table S.3: Size and characteristics of the two datasets.

Datasets	Variables [a]	ALL	Early group	Middle group	Late group	P-value
AD	N	125	35	33	57	—
	Sex	72/28 [b]	60/40	81.8/18.2	73.7/26.3	**0.125**
	Age	84.13±7.23	82.31±8.79	84.85±5.89	84.82±6.8	**0.324**
	PH	6.37±0.28	6.46±0.26	6.33±0.28	6.34±0.28	**0.094**
	PMI	361.02±318.73	416.29±345.81	341.52±327.62	338.37±297.25	0.046
	PLQ_Mn	8.88±8.21	3.71± 4.41	10.7±9.71	11.01±7.77	**<0.000**
	NPrSum	15.01±10.5	7.14±8.57	17.36±10.56	18.47±9.05	**<0.000**
	NTrSum	11.99±8.97	4.37±3.87	11.48±7.52	16.96±8.67	**<0.000**
Datasets	Variables	ALL	Fetal	Postnatal	Adulthood	P-value
Brain development	N	42	20	16	6	—
	Sex	45.2/54.8	50/50	37.5/62.5	50/50	**0.732**
	Age [c]	—	18.25±7.93	6.68±6.61	31.17±7.83	**<0.000**

[a] The full names of the abbreviated variables are the sample size (N), the postmortem interval (PMI), the potential of hydrogen (pH), the plaque density mean (PLQ_Mn), the sum of neuritic plaque density estimates (NPrSum), and the sum of neurofibrillary tangles density estimates (NTrSum).

[b] Categorical variables were represented as frequencies (percentages),continuous variables were expressed as mean ± standard deviation (SD). Chi-square tests or Fisher's precise tests were performed on categorical variables; ANOVA or KW test were carried out on continuous variables, with p<0.05 indicating statistically significant differences.

[c] There are different age measures (PCW and Y) depending on the brain development dataset sample, so it is not appropriate to calculate characteristics for the entire sample.

S2. Analysis of gene heterogeneous expression

Due to gene heterogeneity, the same gene at the same stage may have different expression levels in different brain regions, so the gene may be divided into up-regulated and down-regulated genes at the same time in different brain

regions compared to the previous stage. As discussed in article 2.2.1 for the neutralization phenomenon, we need to make some adjustments to the FC values. Figure S.2 below depicts when the same gene is expressed differently in two and three brain regions, and compares the results of the adjusted FC value compared with the average weighted and unadjusted FC value, where the adjusted FC value is relatively more reflective of the true expression level of the gene in the presence of genetic heterogeneity. It is worth noting that the example here uses FC-based weighting, which is different from the $\log_2 FC$-based weighting method in the paper, but the results are consistent.

Table S.4 and Table S.5 calculate the FC value of A, B and C, D cases in Figure S.2 and the weighted expression genes under different weighting methods, respectively, where FC_{tr} represents the FC value of brain region r in stage t, and y_w represents the weighted gene expression data. Four weighting methods are considered in the table, namely: equal weighting, weighting based on FC, adjusted FC_{up1} and adjusted FC_{up}. The two different adjusted FC value were considered when the weight of up-regulated genes was modified to 1 or kept unchanged, respectively. Using FC value as a measure, for example, when $FC_{down} \times FC_{up} = 1$, we consider that the gene has the same up- and down-regulated effects. Consider the following three scenarios:

- When the effects of up-regulation and down-regulation are basically the same (A in Figure S.2), the results of adjusting FC_{up1} and equal-weight weighting are the same. In comparison, the results based on FC weighting are larger, and the results based on adjusting FC_{up} weighting are smaller.
- When the expression of up-regulated genes was weaker than that of down-regulated genes (B,C in Figure S.2), the results based on equal weight and FC weight were relatively large, while the results based on adjusted FC_{up1} and adjusted FC_{up} were better.
- When the expression of up-regulated genes is stronger than that of down-regulated genes (D in Figure S.2), the results based on equal weight and

FC_{up1} weighting are relatively small, the results based on FC value are significantly larger, and the results based on adjusted FC_{up} are better than those based on adjusted FC_{up}.

In addition, the above did not consider some special cases, for example, in the screening process of differentially expressed genes (DEGs), there may be a situation in which the number of down-regulated genes is significantly more than that of up-regulated genes. In this case, adjusted FC_{up1} can be considered to be modified to adjusted FC_{up0}, and the specific results are shown in section S3.2.

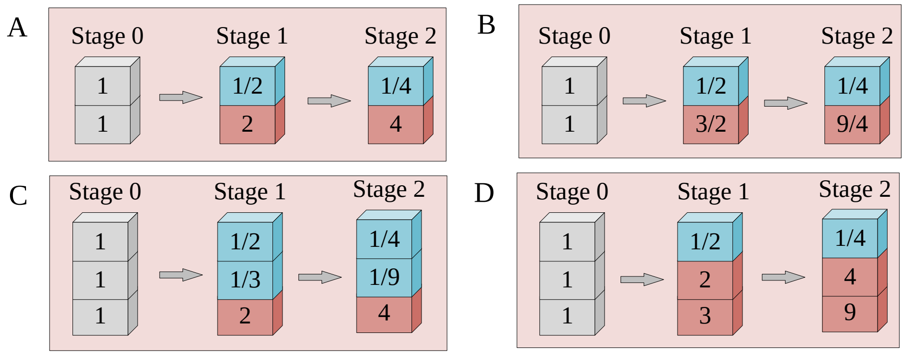

Figure S.2: Illustration of the case with weight adjustment. Only one gene is considered, and the value in each block represents the expression level of the gene, where A, B and C, D are the cases of two brain regions and three brain regions, respectively, and only the gene expression of three stages (Stage 0, Stage 1 and Stage 2) is considered here. According to the calculated FC value, A, B, C and D were divided into three groups: the up-regulation and down-regulation effects were basically the same (a); The expression of up-regulated genes was weaker than that of down-regulated genes (B,C). The expression of up-regulated genes was stronger than that of down-regulated genes (D).

S3. Detailed results for the AD dataset

S3.1. Weighting results for DEGs based on FC values

In this paper, Limma model was used to conduct a preliminary differential gene screen for gene expression levels in different time stages in each brain region. Taking the union of all DEGs from the 19 brain regions, we obtained a total of 1014 DEGs and the results are shown in Figure S.3. Taking the temporal gyrus

Table S.4: The weights of the two brain regions adjust the weighted results.

Case	Weighting	Stage 1			Stage 2		
		FC_{11}	FC_{12}	y_w	FC_{21}	FC_{22}	y_w
A	Equal weight	1.00	1.00	1.250	1.00	1.00	2.125
	FC	0.50	2.00	1.700	0.25	4.00	6.430
	Adjusted FC_{up1}	2.00	1.00	**1.000**	4.00	1.00	**1.000**
	Adjusted FC_{up}	2.00	2.00	**1.250**	4.00	4.00	**2.125**
B	Equal weight	1.00	1.00	1.000	1.00	1.00	1.250
	FC	0.50	1.50	1.250	0.25	2.25	2.050
	Adjusted FC_{up1}	2.00	1.00	**0.833**	4.00	1.00	**0.650**
	Adjusted FC_{up}	2.00	1.50	**0.929**	4.00	2.25	**0.970**

region (region 16) as an example, there were 18431 gene expression data in this region before screening. Differ-ential gene expression analysis was done between each pair of groups (early vs. mid, early vs. late, and mid vs. late) to identify DEGs. The results showed that 48 DEGs (3 upregulated and 45 downregulated) were identified in the comparison between the early-stage and mid-stage groups, while 304 DEGs (13 upregulated and 291 downregulated) were identified in the comparison between the early-stage and late-stage groups, and 37 DEGs (8 upregulated and 29 downregulated) were identified in the comparison between the mid-stage and late-stage groups. After merging of these three sets of DEGs, we got 317 DEGs totally.

S3.2. Comparison of different FC adjustment methods

The reasons for setting different weights for up-regulated and down-regulated genes are shown below, mainly to avoid the problem of positive and negative neutralization after weighting brain regions of the same gene. As shown in Figure S.4, when the number of significantly up-regulated genes is significantly less than the number of significantly down-regulated genes, we kept the weight of significantly upregulated genes constant or set it to 1, which will have no signif-

Table S.5: The weights of the three brain regions adjust the weighted results.

Case	Weighting	Stage 1				Stage 2			
		FC_{11}	FC_{12}	FC_{13}	y_w	FC_{21}	FC_{22}	FC_{23}	y_w
C	Equal weight	1.00	1.00	1.00	1.250	1.00	1.00	1.00	2.125
	FC	0.50	0.33	2.00	0.944	0.25	0.11	4.00	2.787
	Adjusted FC_{up1}	2.00	3.00	1.00	**1.539**	4.00	9.00	1.00	**3.686**
	Adjusted FC_{up}	2.00	3.00	2.00	**0.857**	4.00	9.00	4.00	**1.059**
D	Equal weight	1.00	1.00	1.00	1.830	1.00	1.00	1.00	4.417
	FC	0.50	2.00	3.00	2.409	0.25	4.00	9.00	7.325
	Adjusted FC_{up1}	2.00	1.00	1.00	**1.500**	4.00	1.00	1.00	**2.333**
	Adjusted FC_{up}	2.00	2.00	3.00	**2.000**	4.00	4.00	9.00	**5.768**

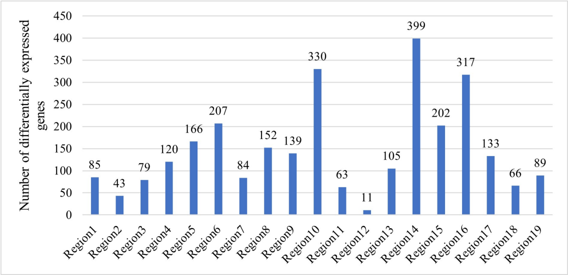

Figure S.3: Number of DEGs in nineteen brain regions

icant effect on the results. Specifically, there were a total of 458 up-regulated genes and 1830 down-regulated genes, which accounts for the main reason for the weight adjustment in the aforementioned methods. Meanwhile, if the weight of insignificant genes is not set to 0, the expression values of those genes that would otherwise be significantly differentially expressed will be ”diluted” by other insignificant DEGs during the weighting process, as shown in the last two panels of Figure S.4. When the unadjusted weights were used for weighting and differential expression analysis, the average expression of significant genes in the late stage patients was higher than that in the early and middle stage patients, showing significant up-regulation, which was contrary to the previous significant down-regulation results, which was unreasonable.

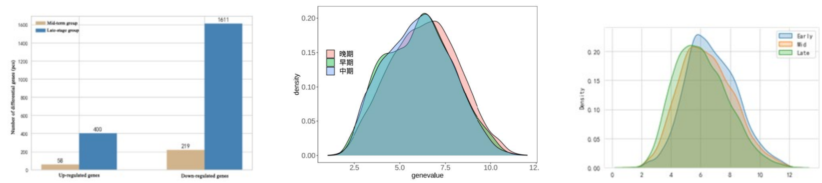

Figure S.4: Illustration of the adjusted weight. The first panel shows the results of the comparison of up-and down-regulated genes in the AD data, mainly comparing the results of early and middle stage, middle and late stage. The latter two panels show show the comparison of results before and after weighting without adjustment of weights.

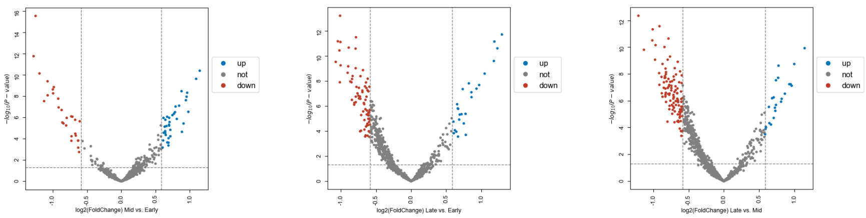

Figure S.5: Differential Gene Expression. The results of the analysis of the DEGs between the weighted gene expression data of three periods.

Differential gene expression analysis was performed on the weighted gene expression data. As shown in Figure S.5.

Because the input features of differentially expressed gene data may present different scales, leading to erroneous results, the scale of the data needs to be standardized in order to prevent these problems. Min-max normalization is one of the main methods to normalize data. Generally, min-max normalization is used to normalize the gene expression value of each gene to the range $[0, 1]$ [2]. However, because of the subsequent need to calculate the log2FC value of the gene data in this paper, a more general min-max normalization formula is considered. That is, the gene expression values are normalized to $[1, a + 1]$. At the same time, in order to choose the appropriate value of a, we also performed the following comparison. As shown in Figure S.6, we compare the gene density curves for different stages with different values of a (2-9) and without normalization (Pre-std.). We observed that the difference in density curves of differentially expressed genes across different stages of AD was maximized

when the value of 'a' was set to 3, providing significant benefits for subsequent analyses.

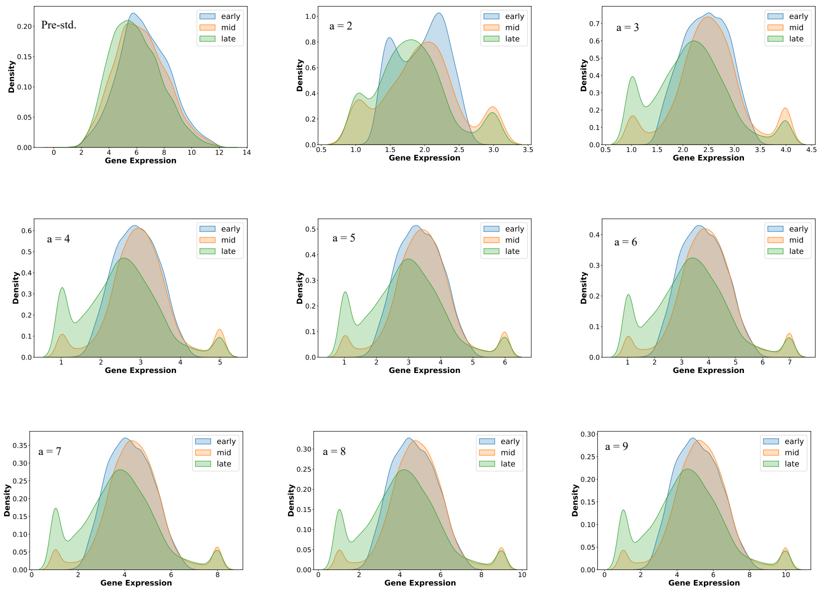

Figure S.6: The density distribution curves of differentially expressed genes in the three stages under various normalization scenarios. 'Pre-std' represents the state before normalization, and 'a' takes values from 2 to 9.

The results of the test based on the comparison of multiple groups are evidently more convincing than intuitive judgment. In our study, we performed pairwise Kolmogorov-Smirnov (KS) tests to assess the distribution of gene expression values across the early, middle, and late stages, both pre-weighted and post-weighted (with 'a' values ranging from 2 to 9). Furthermore, we introduced two additional indicators, namely Maximum Mean Discrepancy (MMD) [3] and Jensen-Shannon Divergence (JS divergence) [4], in order to provide a comprehensive comparison of the results.

Table S.6 provides the indicator statistics values for the pairwise distributions across the three stages. Significantly, both the p-values from the KS test and MMD test are found to be below the threshold of 0.05, indicating a sub-

stantial distinction between the compared distributions. Consequently, we solely present the statistics values for comparison purposes and emphasize the three largest values observed in each column. As depicted in Table 1, when 'a' is set to 3, the results demonstrate either optimal or sub-optimal values for all three indices, at the very least. In conclusion, we posit that the discrepancy in gene expression across different stages can be maximized when utilizing a value of 'a' equal to 3.

Table S.6: Comparison between weighted pre and post distributions

Values	KS statistic			MMD			JS divergence		
	A [a]	B	C	A	B	C	A	B	C
Pre-std.	**0.0713**	0.1460	0.0790	0.0099	0.0184	0.0062	**0.0086**	**0.0930**	**0.0803**
a=2	**0.1364**	0.1740	0.1514	**0.0307**	0.0499	0.0184	**0.0116**	**0.0958**	**0.0832**
a=3	**0.0884**	**0.2259**	**0.2467**	**0.0156**	**0.0674**	**0.0559**	**0.0087**	**0.0832**	**0.0710**
a=4	0.0637	**0.2407**	**0.2565**	**0.0100**	**0.0748**	**0.0583**	0.0073	0.0755	0.0639
a=5	0.0560	**0.2352**	**0.2440**	0.0081	**0.0635**	**0.0554**	0.0066	0.0714	0.0603
a=6	0.0578	0.2220	0.2329	0.0063	0.0589	0.0468	0.0060	0.0689	0.0581
a=7	0.0599	0.2139	0.2268	0.0068	0.0542	0.0451	0.0057	0.0664	0.0560
a=8	0.0629	0.2097	0.2253	0.0093	0.0538	0.0464	0.0053	0.0645	0.0544
a=9	0.0645	0.2064	0.2238	0.0077	0.0577	0.0497	0.0050	0.0627	0.0530

[a] A, B, and C represent the comparison of gene expression distribution between the pairwise combinations of the three stages (early-medium, early-late, medium-late), respectively. 'Pre-std' represents the state before normalization, and 'a' takes values from 2 to 9. We bold the three largest values in each column.

S3.3. The genetic heterogeneity of AD patients

Table S.7 shows the practical results of different FC adjustment methods on AD data, mainly based on the classification accuracy of decision trees for comparison. Among them, because the number of up-regulated genes screened based on AD data was significantly less than the number of down-regulated genes, we considered another way to adjust FC, that is, to set the weight of up-regulated and insignificant genes to 0 (see Method 1 in Table S.7). In addition, we also considered weighting the up-regulated and down-regulated genes separately as a weighting method (see Method 4 in Table S.7), and combined

with the four methods of Adjusted FC_{up1} and Adjusted FC_{up} in Section S2 for comparison.

- Method 1 (Adjusted FC_{up0}): the weight of down-regulated genes was assigned to the negative value of the $\log_2 FC$, while the weight for up-regulated and insignificant genes was set to 0.
- Method 2 (Adjusted FC_{up1}): the weight of down-regulated genes was assigned to the negative value of the $\log_2 FC$, the weight of up-regulated genes as 1, and the weight of insignificant genes as 0.
- Method 3 (Adjusted FC_{up}): the weight of down-regulated genes was assigned to the negative value of the $\log_2 FC$, while keeping the weight of up-regulated genes unchanged and the weight of insignificant genes as 0.
- Method 4 (Adjusted $FC_{up\text{-}down}$): the weight of down-regulated genes was assigned to the negative value of the $\log_2 FC$, kept the weight for up-regulated genes unchanged, set the weight of insignificant genes as 0, and weighted up-regulated and down-regulated genes separately.

As observed from Table S.7, in the AD data, there is a notable imbalance between the number of up-regulated genes and down-regulated genes. Specifically, when gene expression levels are predominantly down-regulated, both Method 1 and Method 2 exhibit slightly better performance. Particularly in terms of classifying early and late stages, Method 1 demonstrates the most favorable outcome. Therefore, in such scenarios, it appears more advantageous to select an appropriate weight adjustment method based on the inherent characteristics of the data itself, which may yield superior results.

S3.4. Introduction to weighting methods.

In addition to using the FC value weighting method to process gene expression data from 19 brain regions, we also experimented with two other methods. One involved ignoring differences in the same gene across different brain regions and using the mean expression value of a patient's gene across all brain regions

Table S.7: Comparison Results.

Stages	Method 1	Method 2	Method 3	Method 4
early vs mid vs late	87.06%	87.06%	89.41%	90.59%
early vs mid	94.00%	88.00%	90.00%	92.00%
early vs late	94.85%	93.18%	89.70%	89.70%
mid vs late	88.33%	93.33%	93.33%	91.67%

as the gene's expression value. The other method involved interpolation to fill in missing gene expression data, treating the same gene in different brain regions as distinct genes for subsequent analysis [5].

Table S.8 provides a comparative evaluation of the proposed spatio-temporal brain region weighting algorithm against two additional approaches, namely brain region averaging and brain region imputation. The results demonstrate that the classification performance attained with the developed algorithm was notably superior to those achieved by the alternative methods, as evidenced by improved accuracy metrics.

Table S.8: Comparison Results.

Comparison	Averaging	Imputation	Weighted
early VS mid VS late	49.41%	47.09%	**88.41**%
early VS mid	70.55%	52.91%	**90.58**%
early VS late	64.24%	51.06%	**88.72**%
mid VS late	63.33%	61.67%	**92.42**%

S3.5. Stability analysis of cluster analysis

Cluster analysis constitutes a pivotal step in the multi-step decision tree methodology. This paper predominantly employs the umap+kmeans method for its implementation, underscoring the significance of investigating the stability of its hyperparameters in influencing subsequent results. Figure S.7 illustrates the impact of UMAP-based dimensionality reduction and changes in kmeans clustering on the classification accuracy of the four decision trees for

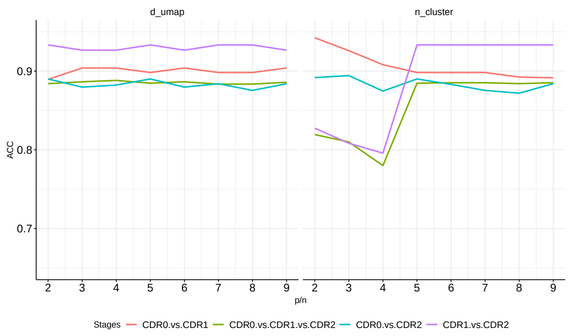

Figure S.7: Stability analysis of cluster analysis. Effects of different dimensionalities in UMAP and varying cluster numbers in K-means on the accuracy (ACC) of multi-step decision tree classification.

the AD dataset. The accuracy values are obtained by averaging over 20 5-fold cross-validations. The left panel demonstrates gene data dimensionality reduction from $n \times p$ to $n_1 \times p$, where n_1 ranges from 2 to 9. Observably, the dimensionality reduction in umap exhibits no pronounced effect on the final result. Meanwhile, the right panel explores the influence of different cluster numbers (umap dimensionality reduction to 5 dimensions), varying the number of clusters from 2 to 9. Notably, for the AD data, clustering genes into 5 categories yields the most effective classification results.

S3.6. Results of other enrichment analyses

Figure S.8 illustrates the enrichment bubble map for GO enrichment analysis of two classes of AD-related differentially expressed gene sets, ClusterB and ClusterD. The enrichment bubble map primarily displays the top 10 functional categories in biological process (BP), cellular component (CC), and molecular function (MF) that are most abundant in the two gene classes. Figure S.9 presents enrichment bar plots and bubble plots from the enrichment analysis of AD, which depict the top 25 most enriched functional categories of DEGs associated with ad in two gene clusters, Cluster B and Cluster D. Additionally, Table S.9 presents the results of enrichment analysis for specific genes within these gene classes. Our analysis focuses on examining the characteristics of the

functional categories in the two gene classes, as well as utilizing select genes to support our findings.

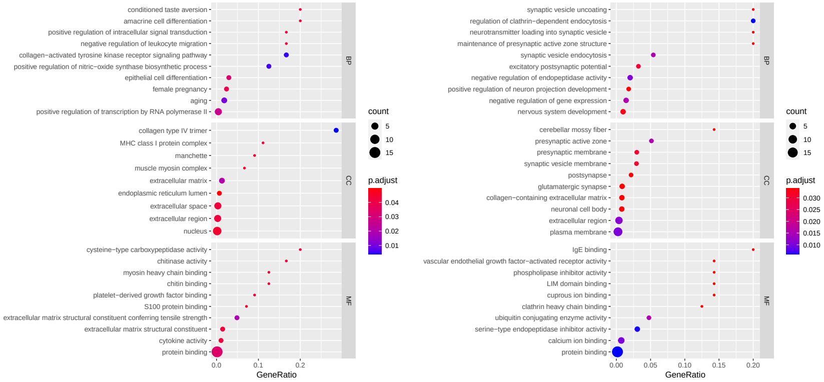

(a) GO Enrichment of ClusterB (b) GO Enrichment of ClusterD

Figure S.8: Bubble graphs depicting the top 10 most enriched functional categories of AD-related DEGs for two gene clusters, Cluster B and Cluster D. The analysis considers different functional categories, including Biological Process (BP), Cellular Component (CC), and Molecular Function (MF), for each cluster.

Table S.9: ClusterB and ClusterD Gene enrichment analysis. Examples of some genes.

Gene classes	GO term	Representative genes	Term	Adj. P-value
ClusterB	GO:0038063	COL4A1, COL4A2	collagen-activated tyrosine kinase receptor signaling pathway	0.0049
	GO:0045944	RORB, NAMPT, IL33, FGF1, FOS	positive regulation of transcription by RNA polymerase II	0.0263
	GO:0016807	AQP1	cysteine-type carboxypeptidase activity	0.0421
	GO:0061304	COL4A1	retinal blood vessel morphogenesis	0.0421
	GO:0004568	CHI3L2	chitinase activity	0.0421
	GO:0034356	NAMPT	NAD biosynthesis via nicotinamide riboside salvage pathway	0.0440
ClusterD	GO:0005515	MEF2C, HPCAL1, VSNL1, ERC2, GABBR2, SNAP9, IPCEF1, SH3GL2	protein binding	0.0060
	GO:2000369	SNAP91, SH3GL2	regulation of clathrin-dependent endocytosis	0.0060
	GO:0048488	SNAP91, SNCB	synaptic vesicle endocytosis	0.0155
	GO:0048786	ERC2, SLC17A7	presynaptic active zone	0.0155
	GO:0030672	SLC17A7, SH3GL2	synaptic vesicle membrane	0.0307
	GO:0048790	ERC2	maintenance of presynaptic active zone structure	0.0340
	GO:1904753	MEF2C	negative regulation of vascular associated smooth muscle cell migration	0.0340
	GO:0005344	IPCEF1	oxygen carrier activity	0.0350
	GO:0005980	PGM2L1	glycogen catabolic process	0.0365
	GO:0045921	VSNL1	positive regulation of exocytosis	0.0401
	GO:0046982	MEF2C, GABBR2	protein heterodimerization activity	0.0428

The enrichment bubble plot of the ClusterB gene class (Figure S.8, Panel (a)) reveals that its biological process (BP) functional categories primarily revolve around the development and synaptic transmission processes of the nervous system, as well as regulatory mechanisms associated with neurotransmitters and

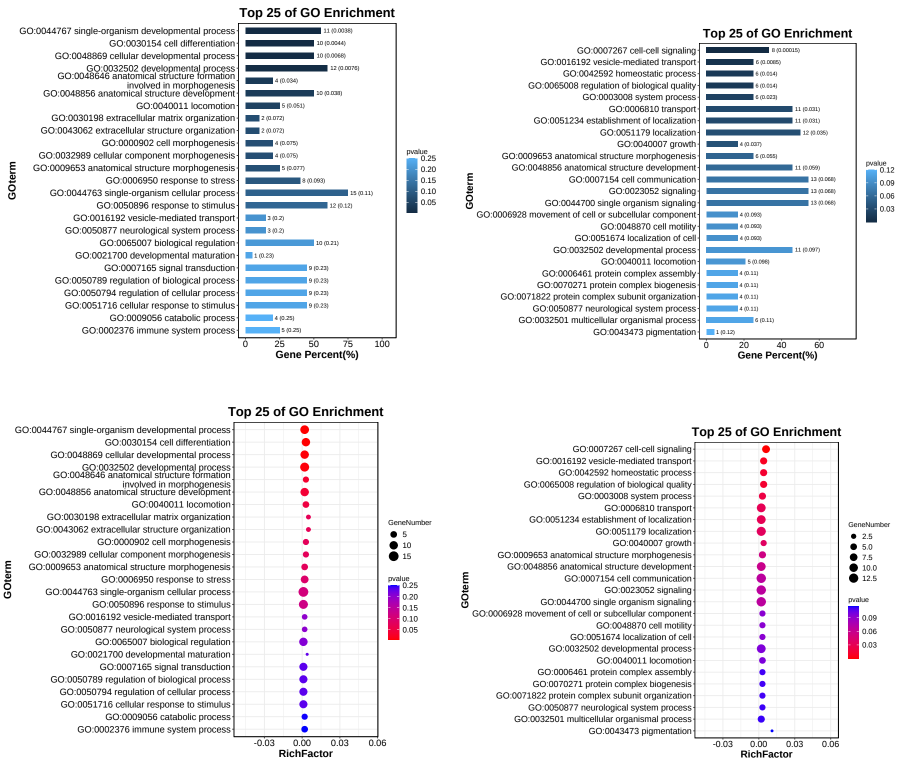

Figure S.9: Enrichment Bar graphs and bubble graphs depicting the top 25 most enriched functional categories of AD-related DEGs for two gene clusters, Cluster B and Cluster D.

synaptic structure. In terms of cellular component (CC) function, the emphasis lies on extracellular structures and intracellular organelles and complexes, including collagen trimers, the extracellular matrix, extracellular space, as well as the nucleus and endoplasmic reticulum within cells. Regarding molecular function (MF), the focus is on the structural components of the extracellular matrix, protein binding, as well as functions related to enzymatic activity and cytokine activity. These functional categories align with the underlying pathological features of AD, specifically the ATN diagnostic framework [6, 7], suggesting the validity of the aforementioned enrichment analysis results.

As previously mentioned, we posit a significant association between the ClusterB gene class and AD patients in late-stage. In the late stages of AD, clinical

manifestations become more pronounced, observable macroscopic alterations in brain biological traits occur, and microscopic protein detection can distinctly identify the advanced symptoms of AD [8]. Based on the results presented here, it is also possible to effectively distinguish patients with moderate-advanced AD by observing related traits controlled by genes in the ClusterB class. For example, [9] showed a potential association between gene AQP1 in astrocytes and Aβ deposition in the AD brain, and senile plaques containing amyloid-beta peptide $A\beta_{1\text{-}42}$ are the major species present in the pathogenesis of AD. Similarly, regulation of genes COL4A1, FOS, and NAMPT had been linked to Aβ deposition and potentially contributed to AD, with COL4A1 and FOS mainly acting through provoking inflammatory reactions [10, 11, 12, 13]. [14] found that a variety of chitinase genes (including gene CHI3L2) were inflammatory biomarkers of AD.

The enrichment bubble plot of the ClusterD gene class (Figure S.8, Panel (b)) reveals that its BP function involves multiple levels such as cell signal transduction and physiological regulation. The CC function focuses on neural cell structure [15], while the MF function involves different types of binding and regulatory activities. In contrast, the functional categories of the ClusterB gene class primarily focus on a certain aspect, whereas the functions of the ClusterD gene class cover a wider range.

Based on the analysis results of the decision tree, we assert a significant association between the ClusterD gene class and AD patients in the early and middle stages. The results of ClusterD enrichment analysis (lower part of Table S.9) revealed a key link between AD and the regulation of clathrin-dependent endocytosis and protein binding, that is, ClusterD class genes that can effectively distinguish between early and middle stage AD patients mainly control protein binding and neuronal synapse and neuronal cell-related metabolic processes. At this time, the clinical features of the patient are not obvious, but the internal metabolism of the brain has undergone microscopic changes [16, 17]. For example, as a neurodegenerative disease affecting cortical regions of the brain, abnormal presynaptic activity is also a potential feature affecting AD. Genes in

the ClusterD class, namely ERC2 and SLC17A7, are associated with presynaptic cell function [18, 19], while SNAP91 and SH3GL2 genes are involved in functions related to synaptic vesicles [20, 21]. In addition, [22] found that cerebral vascular smooth muscle cells were significantly reduced in AD patients, and the enrichment results of ClusterD gene class showed that MEF2C gene was involved in vascular associated smooth muscle cell migration, which verified the findings of this study [23]. [24] found that abnormal glycogen catabolism can also affect neuronal cell metabolism, which represents another potential pathogenic factor in AD, and this result also corresponds to the PGM2L1 gene in Table S.9 [25].

Furthermore, we observed a significant association between oxygen carrier activity (IPCEF1) and protein heterodimerization activity (MEF2C and GABBR2) in both early and middle stages of AD. The formation of dimeric structures by AD-associated genes facilitates synergistic interactions among mutated monomers, leading to the generation of more functional enzyme forms that contribute to the maintenance of brain functionality. Disruption of protein heterodimerization activity associated with MEF2C and GABBR2 genes may compromise this functional stability, ultimately impacting AD progression [26]. Moreover, oxygen carrier activity associated with the IPCEF1 gene influences various aspects of normal cellular activities and represents a potential pathogenic factor in AD [27].

S3.7. Further discussion and analysis of brain heterogeneity

We delve into a comprehensive discussion and comparison of existing studies concerning gene expression heterogeneity in diverse brain regions. Through this exploration, we aim to foster novel perspectives and insights that can guide future research endeavors.

Firstly, the gene heterogeneity examined in this paper primarily pertains to the disparate expression values of the same gene across distinct brain regions, while also considering the temporal heterogeneity in gene expression across early, middle, and late stages. This approach shares similarities with the study conducted by [28], which explores the temporal dimensions and heterogeneity of

diverse cells (brain regions), whereby gene expression heterogeneity intensifies over time. However, notable distinctions exist between the two. In [28], the focus lies on the continuous process of cell division, wherein a clear relationship exists between parent nodes and descendant nodes in the cell lineage tree, and the gene expression data within each cell form a continuous curve. In contrast, the data analyzed in this paper comprise brain slice data obtained from deceased AD patients. Different patients are categorized into three stages: early, middle, and late, and there exists no relationship between "parents" and "offspring" among the samples across the three stages. Acquiring comparable cell clonal expansion data samples for AD-related investigations might pose challenges with current techniques. Consequently, building upon the AD data presented in this paper, we can leverage the two dimensions of brain region and time to scrutinize the heterogeneity of gene expression, thereby fostering a more comprehensive understanding of the patterns of gene expression changes across different brain regions and developmental stages throughout the progression of AD.

-Secondly, there is a need for improvement in the analysis of gene expression heterogeneity addressed in this paper. The current investigation of heterogeneity solely focuses on a single gene, and the weight FC value merely considers the alteration of an individual gene. Moreover, it is worth considering the interplay between gene pairs and the correlation among brain regions (including functional, structural, and spatial adjacency information) when studying gene heterogeneity and determining weight calculations. For instance, [29] associated gene expression heterogeneity with functional and structural networks of brain regions, while also considering gene expression networks. [30] assessed spatial heterogeneity of gene expression by incorporating spatial adjacency relationships, whereas [31] explored how structural discrepancies in distinct brain regions (such as vascular changes) influenced gene expression heterogeneity in AD. These aforementioned analyses rely on valuable data obtained through specific technologies, such as functional magnetic resonance imaging (fMRI) for neuroimaging data and spatial transcriptomics technology for gene expression profiles, which retains the spatial information of tissue sections [32]. Furthermore,

[33] demonstrated the identification of temporal and phenotypic heterogeneity of genotypes using imaging data, enabling effective inference of subtypes and disease stages in neurodegenerative conditions. Exploring gene heterogeneity based on the structure and function of brain regions, as well as the spatial adjacency information among brain regions, along with refining the weight FC calculation in this study, represent promising avenues for future research.

-The heterogeneity of brain regions manifests in diverse manners. On one hand, the heterogeneity is discernible at the intricate single-cell level. Notably, glial cells (e.g., astrocytes, microglia) have recently been demonstrated to exhibit a profound degree of heterogeneity within the same region and across disparate brain regions of the mammalian central nervous system (CNS), encompassing a wide array of phenotypic and functional attributes [34, 35]. On the other hand, the heterogeneity of brain regions in the context of AD is intricately intertwined with genetic and environmental factors. For instance, a study by [36] investigated significant heterogeneity in genetic risk factors (including APOE- 4, APOE- 2, polygenic risk, and familial risk) as well as phenotype-associated traits (such as general health, psychosocial health, and cognitive function) in AD. Furthermore, [37] suggested a potential association between the heterogeneity observed in brain regions affected by AD and demographic factors (such as age, sex, education, and socioeconomic status), as well as genetic factors influencing underlying pathological, cognitive, and behavioral phenotypes.

In summary, there exists a significant avenue for further investigation into the heterogeneity of gene expression in diverse brain regions among patients diagnosed with AD. In addition to exploring the multifaceted dimensions of distinct brain regions and various stages, there is a promising opportunity to effectively acquire an expanded repertoire of images, spatial transcriptome data, and single-cell information from diverse brain regions by synergistically integrating existing technologies, facilitating comprehensive analysis. Furthermore, the heterogeneity of brain regions in AD is intricately linked to genetic and environmental factors, thereby warranting further scholarly inquiry.

Our method not only allows the analysis of gene classes significantly ex-

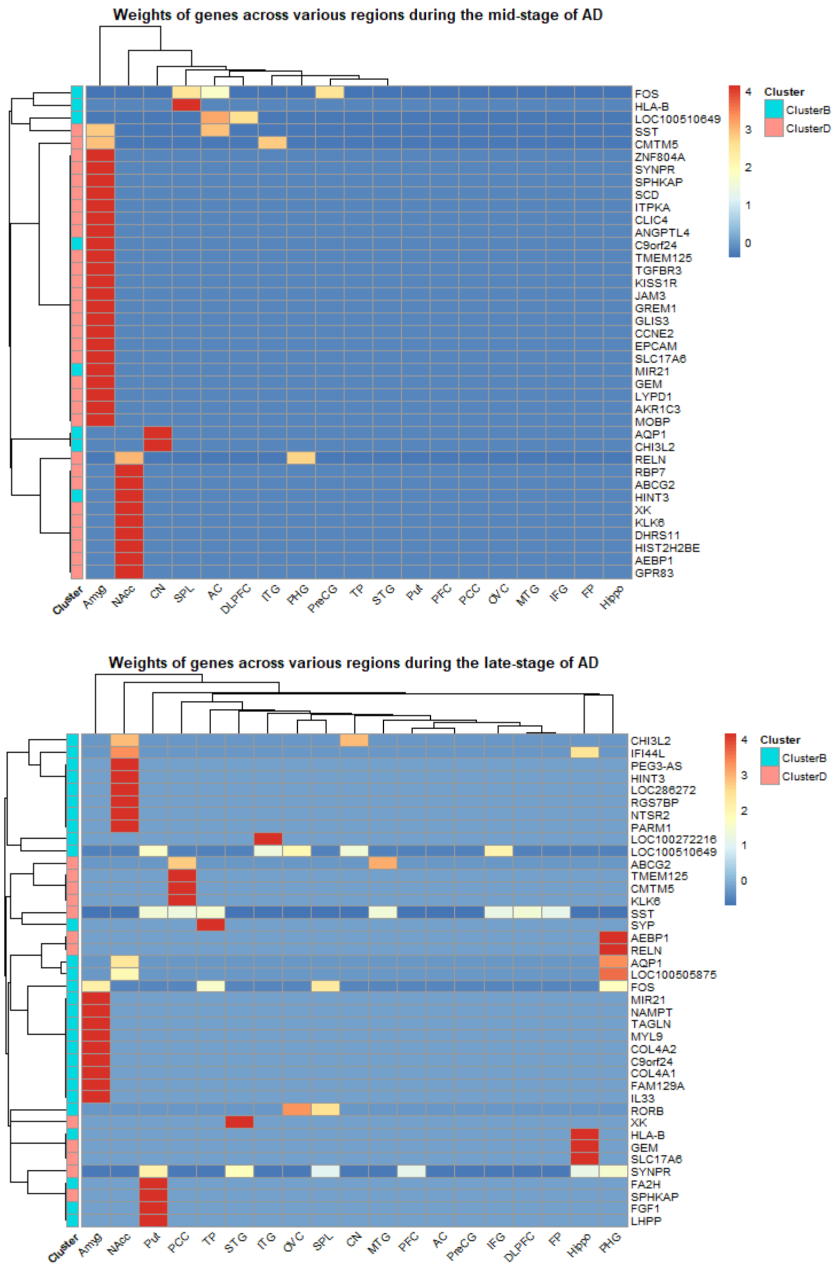

Figure S.10: Clustering heatmap of the adjusted weights of gene classes ClusterB and ClusterD associated with AD in the middle and late stages. The upper and lower panels represent clustering heatmaps with adjusted weights calculated based on early gene expression levels in the middle and late stages, respectively. Each row represents a differentially expressed gene in a specific gene class, and each column corresponds to a different brain region.

pressed in different stages of AD but also facilitates the examination of gene expression differences across various brain regions. In our approach, we assigned weights to the same gene in different brain regions, addressing the issue of gene heterogeneity and enabling the extraction of overall gene expression data for the brain. Subsequently, we investigated significantly expressed gene classes in different stages using multi-step decision trees. Both processes described above can be further studied through reasonable inverse extrapolation. Specifically, we can take the genes from gene classes (such as AD-related gene classes B and D) obtained in the multi-step decision tree step and retroactively incorporate them into the weighting process of the first step. This allows us to obtain weight values for these genes at different stages. The same gene may have varying weights (log2FCad) in different brain regions, where higher weights indicate a greater degree of gene expression changes in the respective brain region. By analyzing these weights, we can directly identify regions of the brain where the gene is significantly expressed, signifying its substantial role in the occurrence and development of AD in those regions.

Figure S.10 displays the clustering heatmap of the adjusted weights for AD-related gene classes ClusterB and ClusterD during the middle and late stages. In this representation, the adjustment weights for the middle and late stages were computed based on gene expression values from the early stages, with normalization applied to the weights of the same gene across different brain regions.

As evident from the clustering heatmap for the middle stage, a majority of the rows correspond to differentially expressed genes in ClusterD, aligning with the conclusion in the paper that suggests a significant association between the ClusterD gene class and early and middle-stage AD patients. Notably, the differential gene expression in ClusterD is primarily observed in the Amygdala (Amyg) and Nucleus Accumbens (Nacc), brain regions that have been consistently implicated in pathological changes related to the early stages of AD, as documented in numerous studies [38, 39, 40]. Similarly, analysis of the clustering heatmap for the late stage reveals more pronounced performance of

differentially expressed genes in ClusterB. Apart from the brain regions Amyg and Nacc, these genes also exhibit significant changes in the Putamen (Put) and Parahippocampal Gyrus (PHG). Previous studies have confirmed significant pathological changes in these relevant brain regions in individuals with AD [41, 42].

Collectively, these results underscore the capability of our method not only to effectively identify genes associated with different stages of AD but also to proficiently characterize changes in gene expression levels across diverse brain regions.

S4. Detailed results for brain development dataset

We selected the top 5% of pairwise log2FC absolute values from each of the three periods as the weighted genes, resulting in a final set of 2,003 DEGs after combining them. Figure S.11 shows the number of DEGs in 16 brain regions, in which it can be seen that the number of up-regulated and down-regulated genes in the three stages of brain development is almost the same.

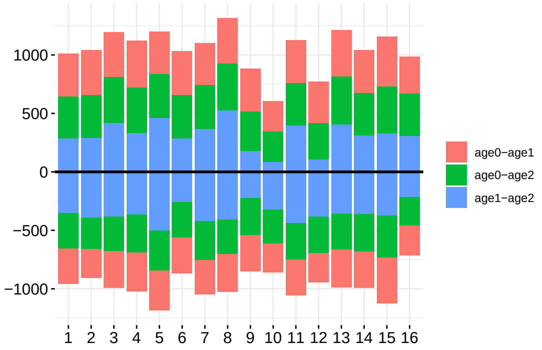

Figure S.11: Bar graph of level grouping of DEGs in 16 brain regions. The bars in the upper and lower halves represent the number of up-regulated and down-regulated genes, respectively. The different colors indicate the DEGs between pairs at various stages of brain development.

S4.1. Analysis results after Weighting

The first panel of Figure S.12 displays the weighted distribution of differential gene expression, which aligns with the observation that gene expression levels in the adult and postnatal development stages are significantly higher compared to those in the fetal development stage. In the multi-step decision tree process, we conducted screening on the weighted data, resulting in the identification of 515 DEGs. The remaining three panels of Figure S.12 present the clustering results of these genes, showcasing the distribution of genes across four classes, along with the variance contribution rate (VCR) of the first principal component. Using the first principal component of the aforementioned four gene classes, we achieved classification accuracies of 89.76%, 92.00%, 95.07%, and 95.07% for distinguishing the three periods and pairwise classifications, respectively. The results of different decision trees are depicted in Figure S.13.

S4.2. Results of enrichment analyses

Based on the decision tree results presented in Figure S.13, we conducted an enrichment analysis for three significant gene classes: ClusterA, ClusterC, and ClusterD. Figure S.14 illustrates a bubble plot representing the enrichment analysis results for these three classes of differentially expressed gene sets associated with brain development. Additionally, Table S.10 displays the outcomes of the enrichment analysis for selected genes within these three gene categories. More detailed results of the enrichment analysis can be found in the attached file (Enrichment_analysis_2datasets.xlsx). Figure S.15 presents enrichment bar plots and bubble plots from the enrichment analysis of brain development dataset, which depict the top 25 most enriched functional categories of DEGs associated with ad in three gene clusters, Cluster A, Cluster C and Cluster D.

The enrichment bubble plot in Figure S.14 clearly demonstrates distinct functional differences among the three gene classes. Regarding BP function, ClusterA is primarily involved in cell signaling pathways and developmental processes. ClusterC is associated with gene expression regulation and cell maturation, exhibiting a broader range of functions. ClusterD predominantly en-

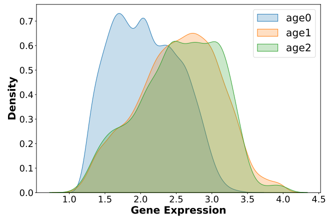

(a) The weighted distribution

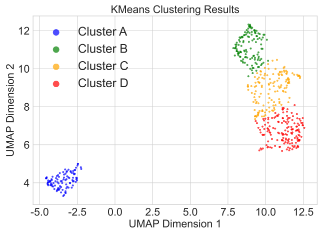

(b) Cluster diagram

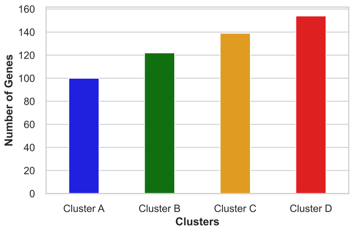

(c) Number of DEGs

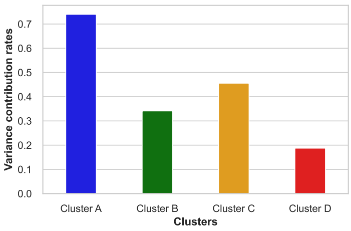

(d) VCR of PCA

Figure S.12: The weighted distribution of differential gene expression and the result of the multi-step decision tree algorithm. The first panel is the weighted gene expression distribution. The second panel is the clustering results. The third panel is the number of DEGs in each category, and the last panel is the VCR of the first principal component after PCA for each cluster gene.

compasses various processes of cell differentiation. In terms of CC function, ClusterA is primarily associated with enveloped body-related structures such as coated nests and small bodies. ClusterC focuses on the nucleus and cytoplasm. ClusterD encompasses a wide range of intracellular and extracellular organelles, exhibiting both breadth and comprehensiveness. Regarding MF function, ClusterA primarily involves the binding of various proteins, exhibiting a single but wide-ranging function. ClusterC primarily involves the combination of RNA and protein, with a narrower scope but in-depth content. ClusterD encompasses binding activity and catalytic activity, with complex functional types. These enriched functions essentially reflect key features of brain development from fetal development to adulthood [43, 44, 45].

In summary, based on the results of the decision tree (Figure S.13), clus-

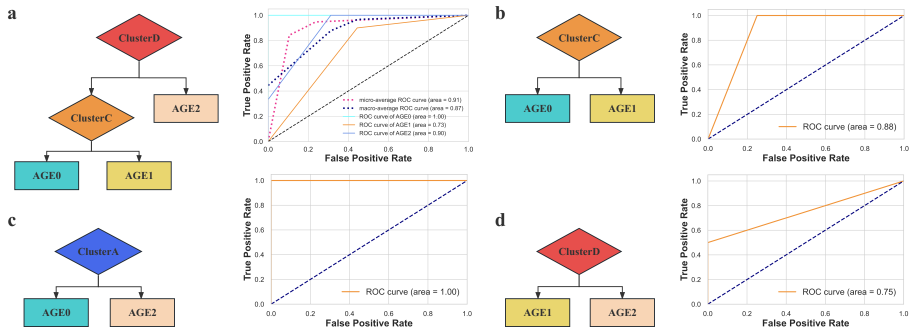

Figure S.13: Fetal development, postnatal development and adulthood decision trees and their classification ROC curves. Among them, diamonds represent taxonomic features (ClusterA, ClusterC and ClusterD), and rectangular blocks (AGE0, AGE1 and AGE2) represent individuals at different periods of brain development.

ter diagram (Figure S.12(b)), and enrichment graphs (Figure S.14), we believe that ClusterA is strongly associated with AGE0, ClusterC is the gene class that distinguishes AGE0 from AGE1, and ClusterD is the key gene class for distinguishing AGE2 from the other two periods.

According to the results of the decision tree (Figure S.13, panel (c)) and the clustering diagram (Figure S.12, panel (b)), ClusterA is associated with AGE0. These genes may be implicated in the healthy development and protection of the fetal brain [46, 47]. For instance, [48] demonstrated the essential role of SOX9 as a determinant of cell fate during embryonic development. Its expression facilitates the differentiation of cells from all three germ layers into various specialized tissues and organs. Dysregulation of Sox9 has been linked to several congenital and acquired diseases. Additionally, [49] identified EPN1 as a crucial gene in uncovering the pathogenesis of medulloblastoma in children. Similarly, [50] pinpointed FNTA as a potential candidate gene for neurodevelopmental disorders (NDD). Furthermore, [51] elucidated that the gene ID3 plays a significant role in cell growth, repair, senescence, apoptotic cell death, and may also contribute to brain microangiopathy.

ClusterC appears to be the gene class that distinguishes AGE0 from AGE1, as illustrated in panel (b) of Figure S.13. These genes might be implicated in the

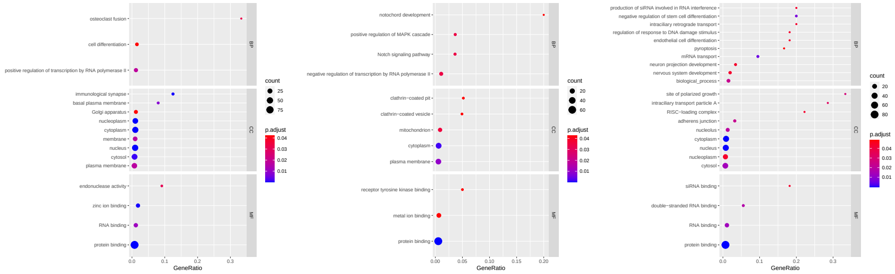

Figure S.14: Bubble graphs depicting the top 10 most enriched functional categories of brain development-related DEGs for three gene clusters, Cluster A, Cluster C and Cluster D. The analysis considers different functional categories, including Biological Process (BP), Cellular Component (CC), and Molecular Function (MF), for each cluster.

Table S.10: ClusterA, ClusterC and ClusterD Gene enrichment analysis. Examples of some genes.

Gene classes	GO term	Representative genes	Term	Adj. P-value
ClusterA	GO:0007219	SOX9,EPN1	Notch signaling pathway	0.034
	GO:0000122	SOX9,ID3	negative regulation of transcription by RNA polymerase II	0.034
	GO:0005905	EPN1	clathrin-coated pit	0.043
	GO:0030971	FNTA	receptor tyrosine kinase binding	0.043
ClusterC	GO:0003725	DHX9, DICER1	double-stranded RNA binding	0.018
	GO:0007399	IGF2BP3	nervous system development	0.044
	GO:0030422	DICER1	production of siRNA involved in RNA interference	0.044
	GO:0070269	DHX9	pyroptosis	0.049
ClusterD	GO:0001772	CORO1A	immunological synapse	<0.001
	GO:0008270	AKR1B1	zinc ion binding	<0.001
	GO:0045944	AKR1B1, APEX1	positive regulation of transcription by RNA polymerase II	0.018
	GO:0004519	APEX1	endonuclease activity	0.031

development of brain region function, where different regions of the brain gradually acquire specific functions, such as language, movement, perception, and so on [52, 53]. For instance, [54] identified heterozygosity for loss-of-function variants in DHX9 as a cause of a novel neurodevelopmental disorder. [55] suggested IGF2BP3 as a potential pathogenic candidate gene for Primary Microcephaly (PM), a condition often associated with mental retardation and other neurodevelopmental defects. In addition, the study conducted by [56] emphasized the significance of DICER1 as a crucial gene involved in miRNA biogenesis. This finding underscores its critical role in early brain patterning and gene regula-

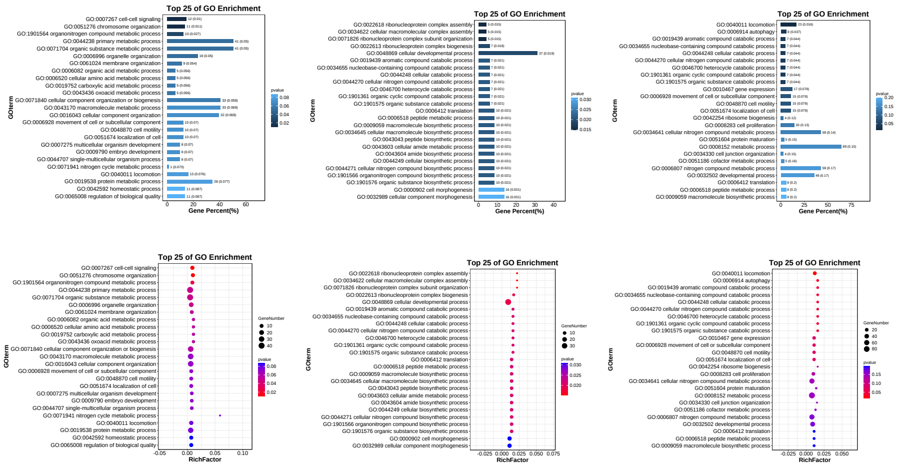

Figure S.15: Enrichment Bar graphs and bubble graphs depicting the top 25 most enriched functional categories of brain development-related DEGs for three gene clusters, Cluster A, Cluster C and Cluster D.

tion. Additionally, [57] proposed that DICER1 plays a vital role in puberty. The deletion of Dicer in Kiss1 cells leads to the development of late-onset hypothalamic hypogonadism in both sexes, with a particular impact on female puberty development and fertility.

ClusterD emerges as the key gene class that distinguishes AGE2 from the other two periods, as depicted in panels (a) and (d) of Figure S.13. During AGE2, brain development reaches a relatively stable and mature stage, yet it can still be influenced by various physiological and environmental factors [58, 59]. For instance, [60] demonstrated that the down-regulation of AKR1B1 gene expression could impede the proliferation, invasion, and migration of glioma cells, ultimately promoting cell apoptosis. This finding suggests a potential role for AKR1B1 in regulating glioma cell behavior. Additionally, [61] investigated the impact of developmental exposure to Pb acetate (PbAc) on hippocampal neurogenesis in rats. The study revealed a significant downregulation of APEX1 gene expression in adulthood, indicating that developmental exposure to PbAc may influence hippocampal neurogenesis through modulation of APEX1 expression. Similarly, [62] indicated that CORO1A plays a crucial role in regulating synap-

tic binding and neurite outgrowth during neurodevelopment. It achieves this by activating the downstream RAC1-PAK1-GPM6A pathway. The downregulation of CORO1A can lead to developmental nervous system damage caused by cadmium (Cd) poisoning.

S5. Comparison of DEGs between the two datasets

The AD and brain development datasets utilized in this study originate from distinct databases, exhibiting dissimilar age distributions. As articulated in the paper's introduction, AD typically manifests in the elderly, while brain development spans from the fetal stage to adulthood and extends into old age. Despite these divergent age profiles, both datasets are gene expression datasets related to the brain. Therefore, this section aims to investigate the disparities in DEGs derived from these two datasets.

In Figure S.16, we compare the DEGs of the two datasets at different steps of the proposed method. Our analysis focuses on three aspects: the comparison of weighted DEGs (Panel (a)), the comparison of DEGs after differential gene screening (Panel (b)), and the comparison of differential genes after multi-step decision trees (Panels (c) and (d)). It is observed that there is some overlap in the DEGs between the two datasets after weighting or differential gene screening (439 and 35 genes are identical). The weighting method, based on $\log_2 FC$ values, considers different brain regions and stages, potentially selecting all possible DEGs. Consequently, the DEGs caused by factors such as aging may not be avoided, so the genes of the two have a large overlap. Upon re-screening with the Limma model, the number of overlapping genes significantly reduces. The weighted gene expression data resolves issues of gene heterogeneity, ensuring that DEGs are more relevant to AD or brain development. After further multi-step decision tree analysis, the genes in the gene classes selected by this step are all genes that are highly associated with different stages of AD or brain development, so there is little overlap between the two (only 6 and 3 genes in common).

In summary, we confidently assert the effectiveness of the proposed method.

It not only efficiently selects gene classes related to different stages of AD but also effectively mitigates the influence of other factors, such as aging.

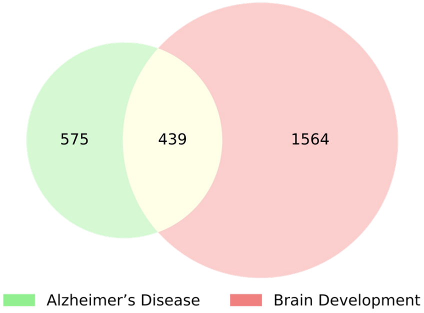

(a) Genes comparison after weighting

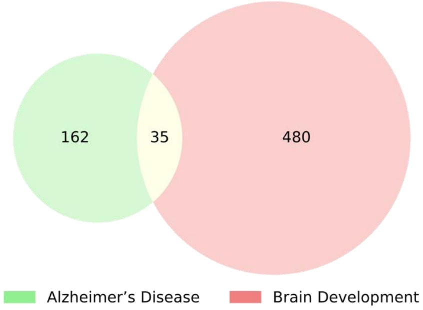

(a) Genes comparison after DE genes screening

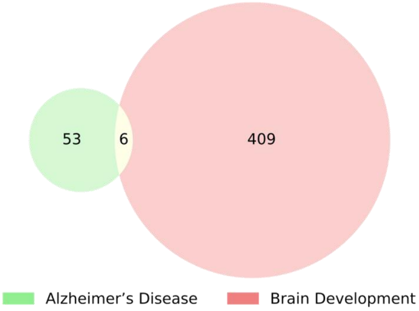

(c) Comparison of DEGs related to AD and brain development

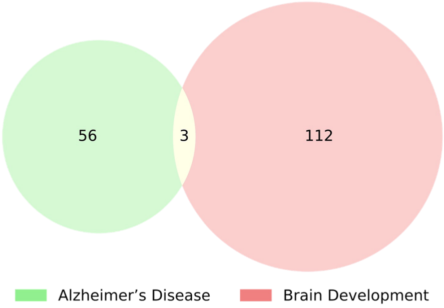

(d) Comparison of DEGs related to AD and brain development in adulthood

Figure S.16: Venn diagram of DEGs for AD and brain development in different methodological steps. Panel (a) displays Venn diagram comparing all DEGs across various brain regions after initial screening based on $\log_2 FC$ values (the weighting method in this paper is also weighted for these genes in different brain regions). Panel (b) presents a comparative Venn diagram of the results of the screening of the weighted DEGs based on the Limma model. Panel (c) presents the Venn diagram of the gene classes associated with different stages of the AD datasets (ClusterB and ClusterD) and brain development datasets (ClusterA, ClusterC, and ClusterD) obtained by the multi-step decision tree approach, respectively. In more detail, panel (d) provides a comparative Venn diagram of gene classes associated with different stages of AD data and gene classes associated with adulthood in brain development data (ClusterD).

References

[1] M. Wang, P. Roussos, A. McKenzie, X. Zhou, Y. Kajiwara, K. J. Brennand, G. C. De Luca, J. F. Crary, P. Casaccia, J. D. Buxbaum, et al., Integrative network analysis of nineteen brain regions identifies molecular signatures and networks underlying selective regional vulnerability to alzheimer's disease, Genome medicine 8 (2016) 1–21.

[2] Y. Wang, Q. Chen, H. Shao, R. Zhang, H. Shen, Generating bulk rna-seq gene expression data based on generative deep learning models and utilizing it for data augmentation, Computers in Biology and Medicine (2023) 107828.

[3] K. M. Borgwardt, A. Gretton, M. J. Rasch, H.-P. Kriegel, B. Schölkopf, A. J. Smola, Integrating structured biological data by kernel maximum mean discrepancy, Bioinformatics 22 (14) (2006) e49–e57.

[4] M. Menéndez, J. Pardo, L. Pardo, M. Pardo, The jensen-shannon divergence, Journal of the Franklin Institute 334 (2) (1997) 307–318.

[5] M. Vysotskiy, X. Zhong, T. W. Miller-Fleming, D. Zhou, N. J. Cox, L. A. Weiss, Integration of genetic, transcriptomic, and clinical data provides insight into 16p11. 2 and 22q11. 2 cnv genes, Genome medicine 13 (1) (2021) 1–26.

[6] C. R. Jack Jr, D. A. Bennett, K. Blennow, M. C. Carrillo, B. Dunn, S. B. Haeberlein, D. M. Holtzman, W. Jagust, F. Jessen, J. Karlawish, et al., Nia-aa research framework: toward a biological definition of alzheimer's disease, Alzheimer's & Dementia 14 (4) (2018) 535–562.

[7] P. Scheltens, B. De Strooper, M. Kivipelto, H. Holstege, G. Chételat, C. E. Teunissen, J. Cummings, W. M. van der Flier, Alzheimer's disease, The Lancet 397 (10284) (2021) 1577–1590.

[8] F. T. Hane, M. Robinson, B. Y. Lee, O. Bai, Z. Leonenko, M. S. Albert, Recent progress in alzheimer's disease research, part 3: diagnosis and treatment, Journal of Alzheimer's disease 57 (3) (2017) 645–665.

[9] T. Misawa, K. Arima, H. Mizusawa, J.-i. Satoh, Close association of water channel aqp1 with amyloid-β deposition in alzheimer disease brains, Acta neuropathologica 116 (2008) 247–260.

[10] V. T. Marchesi, Gain-of-function somatic mutations contribute to inflammation and blood vessel damage that lead to alzheimer dementia: a hypothesis, The FASEB Journal 30 (2) (2016) 503–506.

[11] V. T. Marchesi, Alzheimer's dementia begins as a disease of small blood vessels, damaged by oxidative-induced inflammation and dysregulated amyloid metabolism: implications for early detection and therapy, The FASEB Journal 25 (1) (2011) 5–13.

[12] J. Xu, P. Zhang, Y. Huang, Y. Zhou, Y. Hou, L. M. Bekris, J. Lathia, C.-W. Chiang, L. Li, A. A. Pieper, et al., Multimodal single-cell/nucleus rna sequencing data analysis uncovers molecular networks between disease-associated microglia and astrocytes with implications for drug repurposing in alzheimer's disease, Genome research 31 (10) (2021) 1900–1912.

[13] X. Xie, Y. Gao, M. Zeng, Y. Wang, T.-F. Wei, Y.-B. Lu, W.-P. Zhang, Nicotinamide ribose ameliorates cognitive impairment of aged and alzheimer's disease model mice, Metabolic brain disease 34 (2019) 353–366.

[14] M. Moreno-Rodriguez, S. E. Perez, M. Nadeem, M. Malek-Ahmadi, E. J. Mufson, Frontal cortex chitinase and pentraxin neuroinflammatory alterations during the progression of alzheimer's disease, Journal of Neuroinflammation 17 (2020) 1–15.

[15] M. Otero-Garcia, S. U. Mahajani, D. Wakhloo, W. Tang, Y.-Q. Xue, S. Morabito, J. Pan, J. Oberhauser, A. E. Madira, T. Shakouri, et al.,

Molecular signatures underlying neurofibrillary tangle susceptibility in alzheimer's disease, Neuron 110 (18) (2022) 2929–2948.

[16] A. Porsteinsson, R. Isaacson, S. Knox, M. Sabbagh, I. Rubino, Diagnosis of early alzheimer's disease: clinical practice in 2021, The journal of prevention of Alzheimer's disease 8 (2021) 371–386.

[17] B. Dubois, H. Hampel, H. H. Feldman, P. Scheltens, P. Aisen, S. Andrieu, H. Bakardjian, H. Benali, L. Bertram, K. Blennow, et al., Preclinical alzheimer's disease: definition, natural history, and diagnostic criteria, Alzheimer's & Dementia 12 (3) (2016) 292–323.

[18] M. Martinez, V. I. Torres, C. P. Vio, N. C. Inestrosa, Canonical wnt signaling modulates the expression of pre-and postsynaptic components in different temporal patterns, Molecular Neurobiology 57 (2020) 1389–1404.

[19] S. Sragovich, A. Malishkevich, Y. Piontkewitz, E. Giladi, O. Touloumi, R. Lagoudaki, N. Grigoriadis, I. Gozes, The autism/neuroprotection-linked adnp/nap regulate the excitatory glutamatergic synapse, Translational psychiatry 9 (1) (2019) 2.

[20] M. Nguyen, Y. C. Wong, D. Ysselstein, A. Severino, D. Krainc, Synaptic, mitochondrial, and lysosomal dysfunction in parkinson's disease, Trends in Neurosciences 42 (2) (2019) 140–149.

[21] R.-t. Hu, Q. Yu, S.-d. Zhou, Y.-x. Yin, R.-g. Hu, H.-p. Lu, B.-l. Hu, Co-expression network analysis reveals novel genes underlying alzheimer's disease pathogenesis, Frontiers in Aging Neuroscience 12 (2020) 605961.

[22] T.-T. Zhang, Q.-Q. Lei, J. He, X. Guan, X. Zhang, Y. Huang, Z.-Y. Zhou, R.-X. Fan, T. Wang, C.-X. Li, et al., Bestrophin3 deficiency in vascular smooth muscle cells activates mekk2/3–mapk signaling to trigger spontaneous aortic dissection, Circulation (2023).

[23] R. Maisuria, A. Norton, C. Shao, E. W. Bradley, K. Mansky, Conditional loss of mef2c expression in osteoclasts leads to a sex-specific osteopenic phenotype, International Journal of Molecular Sciences 24 (16) (2023) 12686.

[24] E. Byman, N. Schultz, A. M. Blom, M. Wennström, et al., A potential role for α-amylase in amyloid-β-induced astrocytic glycogenolysis and activation, Journal of Alzheimer's Disease 68 (1) (2019) 205–217.

[25] E. Morava, U. A. Schatz, P. M. Torring, M.-A. Abbott, M. Baumann, C. Brasch-Andersen, N. Chevalier, U. Dunkhase-Heinl, M. Fleger, T. B. Haack, et al., Impaired glucose-1, 6-biphosphate production due to bi-allelic pgm2l1 mutations is associated with a neurodevelopmental disorder, The American Journal of Human Genetics 108 (6) (2021) 1151–1160.

[26] J. C. Udeochu, S. Amin, Y. Huang, L. Fan, E. R. S. Torres, G. K. Carling, B. Liu, H. McGurran, G. Coronas-Samano, G. Kauwe, et al., Tau activation of microglial cgas–ifn reduces mef2c-mediated cognitive resilience, Nature neuroscience (2023) 1–14.

[27] M. Huang, Y. Yu, W. Yang, Q. Feng, A. D. N. Initiative, Incorporating spatial–anatomical similarity into the vgwas framework for ad biomarker detection, Bioinformatics 35 (24) (2019) 5271–5280.

[28] F. Chen, Z. Li, X. Zhang, P. Wu, W. Yang, J. Yang, X. Chen, J.-R. Yang, Phylogenetic comparative analysis of single-cell transcriptomes reveals constrained accumulation of gene expression heterogeneity during clonal expansion, Molecular Biology and Evolution 40 (5) (2023) msad113.

[29] W. Liu, L.-L. Zeng, H. Shen, Z.-T. Zhou, D. Hu, Functional orderly topography of brain networks associated with gene expression heterogeneity, Communications Biology 5 (1) (2022) 1083.

[30] B. F. Miller, D. Bambah-Mukku, C. Dulac, X. Zhuang, J. Fan, Characterizing spatial gene expression heterogeneity in spatially resolved single-cell

transcriptomic data with nonuniform cellular densities, Genome research 31 (10) (2021) 1843–1855.

[31] A. Bryant, Z. Li, R. Jayakumar, A. Serrano-Pozo, B. Woost, M. Hu, M. E. Woodbury, A. Wachter, G. Lin, T. Kwon, et al., Endothelial cells are heterogeneous in different brain regions and are dramatically altered in alzheimer's disease, Journal of Neuroscience 43 (24) (2023) 4541–4557.

[32] E. Gerrits, Y. Heng, E. W. Boddeke, B. J. Eggen, Transcriptional profiling of microglia; current state of the art and future perspectives, Glia 68 (4) (2020) 740–755.

[33] A. L. Young, R. V. Marinescu, N. P. Oxtoby, M. Bocchetta, K. Yong, N. C. Firth, D. M. Cash, D. L. Thomas, K. M. Dick, J. Cardoso, et al., Uncovering the heterogeneity and temporal complexity of neurodegenerative diseases with subtype and stage inference, Nature communications 9 (1) (2018) 4273.

[34] I. Matias, J. Morgado, F. C. A. Gomes, Astrocyte heterogeneity: impact to brain aging and disease, Frontiers in aging neuroscience 11 (2019) 59.

[35] Y.-L. Tan, Y. Yuan, L. Tian, Microglial regional heterogeneity and its role in the brain, Molecular psychiatry 25 (2) (2020) 351–367.

[36] H. M. Wu, A. M. Goate, P. F. O'Reilly, Heterogeneous effects of genetic risk for alzheimer's disease on the phenome, Translational Psychiatry 11 (1) (2021) 406.

[37] R. Duara, W. Barker, Heterogeneity in alzheimer's disease diagnosis and progression rates: implications for therapeutic trials, Neurotherapeutics 19 (1) (2023) 8–25.

[38] C. Guo, D. Wen, Y. Zhang, R. Mustaklem, B. Mustaklem, M. Zhou, T. Ma, Y.-Y. Ma, Amyloid-β oligomers in the nucleus accumbens decrease motivation via insertion of calcium-permeable ampa receptors, Molecular psychiatry 27 (4) (2022) 2146–2157.

[39] A. Cordella, P. Krashia, A. Nobili, A. Pignataro, L. La Barbera, M. T. Viscomi, A. Valzania, F. Keller, M. Ammassari-Teule, N. B. Mercuri, et al., Dopamine loss alters the hippocampus-nucleus accumbens synaptic transmission in the tg2576 mouse model of alzheimer's disease, Neurobiology of disease 116 (2018) 142–154.

[40] K. M. Stouffer, X. Grande, E. Duezel, M. Johansson, B. Creese, M. P. Witter, M. I. Miller, L. E. Wisse, D. Berron, Amidst an amygdala renaissance in alzheimer's disease, Brain (2023) awad411.

[41] P. M. Cogswell, H. J. Wiste, M. L. Senjem, J. L. Gunter, S. D. Weigand, C. G. Schwarz, A. Arani, T. M. Therneau, V. J. Lowe, D. S. Knopman, et al., Associations of quantitative susceptibility mapping with alzheimer's disease clinical and imaging markers, Neuroimage 224 (2021) 117433.

[42] L. Krajcovicova, P. Klobusiakova, I. Rektorova, Gray matter changes in parkinson's and alzheimer's disease and relation to cognition, Current Neurology and Neuroscience Reports 19 (2019) 1–9.

[43] S. Gulsuner, T. Walsh, A. C. Watts, M. K. Lee, A. M. Thornton, S. Casadei, C. Rippey, H. Shahin, D. Braff, K. S. Cadenhead, et al., Spatial and temporal mapping of de novo mutations in schizophrenia to a fetal prefrontal cortical network, Cell 154 (3) (2013) 518–529.

[44] M. Edde, G. Leroux, E. Altena, S. Chanraud, Functional brain connectivity changes across the human life span: From fetal development to old age, Journal of neuroscience research 99 (1) (2021) 236–262.

[45] E. A. Rietman, S. Taylor, H. T. Siegelmann, M. A. Deriu, M. Cavaglia, J. A. Tuszynski, Using the gibbs function as a measure of human brain development trends from fetal stage to advanced age, International Journal of Molecular Sciences 21 (3) (2020) 1116.

[46] S. Peyvandi, C. Rollins, Fetal brain development in congenital heart disease, Canadian Journal of Cardiology 39 (2) (2023) 115–122.

[47] L. Li, C. L. Maire, M. Bilenky, A. Carles, A. Heravi-Moussavi, C. Hong, A. Tam, B. Kamoh, S. Cho, D. Cheung, et al., Epigenomic programming in early fetal brain development, Epigenomics 12 (12) (2020) 1053–1070.

[48] A. Jo, S. Denduluri, B. Zhang, Z. Wang, L. Yin, Z. Yan, R. Kang, L. L. Shi, J. Mok, M. J. Lee, et al., The versatile functions of sox9 in development, stem cells, and human diseases, Genes & diseases 1 (2) (2014) 149–161.

[49] P. Huang, Y.-D. Guo, H.-W. Zhang, Identification of hub genes in pediatric medulloblastoma by multiple-microarray analysis, Journal of Molecular Neuroscience 70 (2020) 522–531.

[50] N. Nassir, I. Sati, S. Al Shaibani, A. Ahmed, O. Almidani, H. Akter, M. Woodbury-Smith, A. A. Tayoun, M. Uddin, A. Albanna, Detection of copy number variants and genes by chromosomal microarray in an emirati neurodevelopmental disorders cohort, neurogenetics 23 (2) (2022) 137–149.

[51] C. M. Perez, Q. Felty, Molecular basis of the association between transcription regulators nuclear respiratory factor 1 and inhibitor of dna binding protein 3 and the development of microvascular lesions, Microvascular Research 141 (2022) 104337.

[52] N. Vijayakumar, Z. O. de Macks, E. A. Shirtcliff, J. H. Pfeifer, Puberty and the human brain: Insights into adolescent development, Neuroscience & Biobehavioral Reviews 92 (2018) 417–436.

[53] R. Mychasiuk, G. A. Metz, Epigenetic and gene expression changes in the adolescent brain: What have we learned from animal models?, Neuroscience & Biobehavioral Reviews 70 (2016) 189–197.

[54] M. Yamada, Y. Nitta, T. Uehara, H. Suzuki, F. Miya, T. Takenouchi, M. Tamura, S. Ayabe, A. Yoshiki, A. Maeno, et al., Heterozygous loss-of-function dhx9 variants are associated with neurodevelopmental disorders: Human genetic and experimental evidences, European Journal of Medical Genetics (2023) 104804.

[55] S. Duerinckx, J. Désir, C. Perazzolo, C. Badoer, V. Jacquemin, J. Soblet, I. Maystadt, Y. Tunca, B. Blaumeiser, B. Ceulemans, et al., Phenotypes and genotypes in non-consanguineous and consanguineous primary microcephaly: High incidence of epilepsy, Molecular genetics & genomic medicine 9 (9) (2021) e1768.

[56] A. Prieto-Colomina, V. Fernández, K. Chinnappa, V. Borrell, Mirnas in early brain development and pediatric cancer: At the intersection between healthy and diseased embryonic development, Bioessays 43 (7) (2021) 2100073.

[57] J. Roa, M. Ruiz-Cruz, F. Ruiz-Pino, R. Onieva, M. J. Vazquez, M. J. Sanchez-Tapia, J. M. Ruiz-Rodriguez, V. Sobrino, A. Barroso, V. Heras, et al., Dicer ablation in kiss1 neurons impairs puberty and fertility preferentially in female mice, Nature Communications 13 (1) (2022) 4663.

[58] U. A. Tooley, D. S. Bassett, A. P. Mackey, Environmental influences on the pace of brain development, Nature Reviews Neuroscience 22 (6) (2021) 372–384.

[59] P. S. Hüppi, Growth and development of the brain and impact on cognitive outcomes, Importance of Growth for Health and Development 65 (2010) 137–151.

[60] L. Luo, C. Chen, H. He, M. Cai, C. Ling, Silencing of long non-coding rna (lncrna) non-coding rna activated by dna damage (norad) inhibits proliferation, invasion, migration, and promotes apoptosis of glioma cells via downregulating the expression of akr1b1, Medical Science Monitor: International Medical Journal of Experimental and Clinical Research 26 (2020) e922659–1.

[61] R. Yamashita, Y. Takahashi, K. Takashima, H. Okano, R. Ojiro, Q. Tang, S. Kikuchi, M. Kobayashi, B. Ogawa, M. Jin, et al., Induction of cellular senescence as a late effect and bdnf-trkb signaling-mediated ameliorating

effect on disruption of hippocampal neurogenesis after developmental exposure to lead acetate in rats, Toxicology 456 (2021) 152782.

[62] J. Feng, S. Chen, Y. Wang, Q. Liu, M. Yang, X. Li, C. Nie, J. Qin, H. Chen, X. Yuan, et al., Maternal exposure to cadmium impairs cognitive development of male offspring by targeting the coronin-1a signaling pathway, Chemosphere 225 (2019) 765–774.
